# Preclinical efficacy and safety evaluation of BD211 autologous CD34^+^ hematopoietic stem cell injection for transfusion-dependent β-thalassemia in NCG-X mice

**DOI:** 10.3389/fcell.2025.1607707

**Published:** 2025-06-16

**Authors:** Xuedong Dai, Zike Li, Shuang Chen, Ying Huang, Sikai Ling, Quanjun Wang, Qi Wang

**Affiliations:** ^1^ Department of Toxicology, School of Public Health, Peking University, Beijing, China; ^2^ SAFE Pharmaceutical Technology Co., Ltd., Beijing, China; ^3^ BDGENE Therapeutics Co. Ltd., BDGENE Therapeutics, Shanghai, China

**Keywords:** transfusion-dependent β-thalassemia, BD211, NCG-X mice, no-observed-adverseeffect level, toxicokinetic, distribution

## Abstract

**Introduction:**

Autologous CD34^+^ hematopoietic stem cell-based therapies have shown promise in addressing therapeutic needs. However, a comprehensive evaluation of their efficacy and safety is crucial before clinical application. This study aimed to assess the efficacy and safety profile of BD211 autologous CD34^+^ hematopoietic stem cell injection in NCG-X mice.

**Methods:**

NCG-X mice were administered BD211 intravenously at doses of 4.0 × 10^5^ and 1.2 × 10^6^ cells per mouse, followed by withdrawal and observation for 13 weeks. Efficacy was evaluated by monitoring the engraftment and differentiation of BD211 into human erythroid cells within the mouse bone marrow and blood. Safety was assessed through clinical observation, pathology, organ weight measurements, and histopathology. Toxicokinetic studies and distribution of BD211 were determined via validated quantitative PCR.

**Results:**

Mortality was observed in all groups of mice with no correlation to dose or BD211. No abnormal effects related to BD211 administration on clinical responses, body weight, or food intake were observed. BD211 successfully engrafted and differentiated into human erythroid cells within the mouse bone marrow and blood.

**Conclusion:**

The no observed adverse effect level of BD211 was established at 1.2 × 10^6^ cells per mouse. BD211 shows potential as a safe therapeutic approach for treating transfusion-dependent thalassemia.

## 1 Introduction

Transfusion-dependent β-thalassemia (TDT) is among the most prevalent monogenic disorders globally, with a particularly high incidence in the Eastern Mediterranean, Southeast Asia, and southern China (Weatherall, 2010; Thong and Ngim, 2021). Approximately 60,000 new cases are diagnosed annually. TDT arises from mutations in the β-globin gene (HBB), leading to diminished synthesis (β+) or complete absence (β0) of functional β-globin (Boulad et al., 2022; Galanello and Origa, 2010). The aberrant production of β-globin disrupts the equilibrium between β-globin and α-globin chains, resulting in the aggregation of surplus unpaired α-globin chains and the formation of precipitates (Li et al., 2024; Chinese Medical Association, 2022). This process damages the red blood cell membrane and induces intravascular hemolysis (Cappellini et al., 2014; Lang and Lang, 2015). For individuals with TDT, conventional treatments encompass lifelong red blood cell transfusions and periodic iron chelation therapy (Shah et al., 2019; Origa, 2017; Cappellini et al., 2011). Although allogeneic hematopoietic stem cell transplantation (HSCT) from human leukocyte antigen (HLA)-matched donors offer a cure for TDT, its application is constrained by the limited availability of suitable donors and the associated risks of transplantation, including graft failure and graft-versus-host disease (Yang et al., 2024; Kleinschmidt et al., 2025).

Advances in scientific technology have facilitated significant progress in gene therapies for TDT (Brendel et al., 2020; Malay et al., 2024). In May 2019, Bluebird Bio’s Zynteglo received conditional approval in the European Union, marking it the world’s first gene therapy for TDT (Locatelli et al., 2022). Numerous gene therapies are currently undergoing clinical investigation (Segura et al., 2023; Magrin et al., 2022; Wattanapanitch et al., 2018; Zeng et al., 2020). However, β0/β0 thalassemia represents the most severe form of TDT and poses a significant challenge for gene therapy. The efficacy of existing gene therapies for β^0^/β^0^ thalassemia still requires further investigation to be fully validated (Thompson et al., 2018). Against this backdrop, BD211 autologous CD34^+^ hematopoietic stem cell injection (BD211) has emerged as a promising therapeutic approach. BD211 uses an optimized lentiviral vector system to deliver the β^A−T87Q^-globin gene into patient-derived hematopoietic stem cells. The product’s distinctive technological advantages include (1) a proprietary insulator design integrated into the lentiviral vector architecture to minimize potential insertional mutagenesis risks and (2) an optimized hemoglobin subunit beta (HBB) gene expression cassette engineered to achieve physiological hemoglobin levels comparable to those observed in healthy individuals. In preliminary studies, BD211 was successfully administered to two patients with severe β0/β0 thalassemia. A 2-year follow-up demonstrated its excellent safety and significant efficacy, bringing new hope and promise to the gene therapy of TDT (Li et al., 2024).

In this study, a single-dose toxicity test of BD211 was conducted via intravenous injection in NCG-X mice, followed by a 13-week recovery period. The aim was to assess the nature, severity, dose-response and time-effect relationships, and reversibility of potential toxic reactions induced by the test substance. Additionally, this study aimed to identify target organs or tissues of toxicity and conduct concurrent studies on oncogenicity, toxicokinetics, and tissue distribution. This research provides valuable animal experimental data for clinical research and highlights key indicators that must be closely monitored in clinical practice.

## 2 Materials and methods

### 2.1 Cells and *in vitro* erythroid differentiation

The BD211 autologous CD34^+^ hematopoietic stem cell injection, non-transduced stem cell, and its cryopreservation solution were sourced from BDGENE Therapeutics Co. Ltd. Human CD34^+^ hematopoietic stem cells were isolated from the peripheral blood of healthy donors following Filgrastim (Recombinant Human Granulocyte Colony-stimulating Factor) mobilization. On the day of use, the BD211 cells and non-transduced stem cells were thawed and maintained on ice until transplantation, with the entire transplantation procedure completed within 4 h after cell thawing. Viability was assessed using the trypan blue exclusion assay, with 78%–90% of the cells demonstrating viability. The hematopoietic differentiation potential and target gene expression of BD211 were confirmed *in vitro* through flow cytometry and PCR analysis (Figure 2).

### 2.2 Animals

Specific pathogen-free (SPF) grade NCG-X mice, evenly categorized between males and females and aged 7–9 weeks, were procured from Jiangsu Jicui Yaokang Biotechnology Co., Ltd. (GemPharmatech Co., Ltd., Nanjing, China). The mice were housed in individually ventilated cages, with the animal room temperature maintained at 22.0°C–26.0°C and relative humidity controlled at 40%–70%. The central air conditioning system provided ventilation at a rate of ≥15 times per hour, and the lighting was set to a 12-h light/12-h dark cycle. Cage ventilation was set at 30–80 times per hour. The facilities used in this study have been accredited by the Association for Assessment and Accreditation of Laboratory Animal Care. All experimental animals were bred and used for scientific research purposes. All procedures were reviewed and approved by the Institutional Animal Care and Use Committee of Guoke Saifu Hebei Pharmaceutical Technology Co., Ltd. (approval numbers: IACUC-2023-149 and IACUC-2023-220).

### 2.3 Experimental design

This study is part of the preclinical safety evaluation of the BD211 autologous CD34^+^ hematopoietic stem cell injection for treating TDT. The study design adhered to ICH guidelines M3 (R2) and relevant technical guidelines (M3R2 I, 2009; National Medical Products Administration, 2017; National Medical Products Administration, 2021).

Overall, 222 SPF-grade NCG-X mice, aged 7–9 weeks, were randomly assigned to four groups, each with an equal number of males and females. The groups included a vehicle control group (n = 40) that received 0.4 mL of Biolife Solutions CS10 cell cryopreservation solution, a mock control group (n = 52) that received 0.4 mL of non-transduced CD34^+^ hematopoietic stem cells (3 × 10^6^ cells/mL), and BD211 low-dose (n = 52) and high-dose groups (n = 78) that received 0.13 mL and 0.4 mL of BD211 (3 × 10^6^ cells/mL), respectively. The administered doses were 0, 1.2 × 10^6^, 4.0 × 10^5^, and 1.2 × 10^6^ cells per mouse, equivalent to 0, 6.0 × 10^7^, 2.0 × 10^7^, and 6.0 × 10^7^ cells/kg, respectively. Based on the results of previous studies, the low dose was considered the effective dose, while the high dose was anticipated to induce adverse reactions. Mice were administered a single intravenous injection via the tail vein. The first day of administration was designated as Day 1 (D1), and the mice were observed continuously for 13 weeks (D92).

**FIGURE 1:**
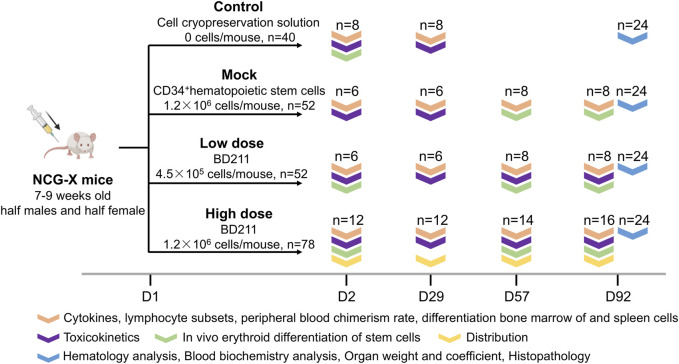
Overview of the study design. Experimental groups: Control (vehicle solution), Mock (non-transduced CD34^+^ hematopoietic stem cells), Low-dose (BD211, 4.5 × 10^5^ cells/mouse), High-dose (BD211, 1.2 × 10^6^ cells/mouse). D: day post-transplantation; n: number (biological replicates).

Evaluations were conducted at multiple time points to assess various parameters. Throughout the experimental period, daily observations were made of the general condition of the mice. Body weight and food intake were measured weekly, and ophthalmological examinations were conducted on D0 and D90. On D2 (24 h post-administration), D57, and D92, assessments focused on *in vivo* erythroid differentiation of stem cells, including the differentiation efficiency of hCD235a^+^ and hCD71^+^ cells, along with the expression levels of the β^A−T87Q^-globin target gene. Additionally, a more comprehensive series of analyses were performed on D2, D29, D57, and D92, encompassing stem cell stemness through lymphocyte and lymphocyte subset profiling, measurement of reconstitution efficiency in mouse, and cellular differentiation ratios in peripheral blood, spleen, and bone marrow. The analyses also included cytokine level analysis (IFN-γ, TNF-α, IL-2, IL-4, IL-6, and IL-10), tissue distribution (BD211 high-dose group only), vector copy number (VCN) quantification, and toxicokinetic parameters, including BD211 levels based on Woodchuck Hepatitis Virus Post-transcriptional Regulatory Element (WPRE) gene concentration and expression analysis of both β-globin and β^A−T87Q^-globin genes. The solvent and mock control groups were tested only on D2 and D29. On D92, hematological, blood biochemical, gross dissection, organ weighing, bone marrow smear examination, histopathological examination, and oncogenicity assessment were performed (Figure 1).

Manufacturer details for reagents and instruments are listed in Supplementary Tables S5, 6.

### 2.4 Clinical examination

During the trial period, daily observations were conducted to record the appearance, dosing site, behavior, skin condition, respiration, mouth and nose, glandular secretion, fecal and urinary characteristics, and any instances of death. Body weight and food intake were measured weekly. Ophthalmic examinations were performed the day before administration and on D90, with a necropsy conducted on D92. Hematological analyses (including coagulation), biochemical analyses (including electrolytes), and histopathological examinations were performed. Gross dissections were carried out, and major organs were weighed.

### 2.5 Detection of VCN

Genomic DNA for VCN analysis was extracted from BD211-transduced CD34^+^ cells cultured for 14 days post-transduction, and whole blood and bone marrow samples were collected from NCG-X mice on D2, D29, D57, and D92 post-administration. Dual fluorescent quantitative PCR was performed using a real-time PCR instrument (Thermo Fisher Scientific, Quant Studio 5) to amplify the viral element WPRE and the human cellular reference gene RNase P using the primers and probe sequences provided in Supplementary Table S4. Thermocycling conditions were as follows: 37°C for 2 min, 95°C for 5 min, followed by 45 cycles of 95°C for 15 s and 60°C for 40 s. Fluorescence signals were monitored during the 60°C phase through the FAM (WPRE) and VIC (RNAseP) channels. WPRE-containing vector RNA undergoes reverse transcription during transduction and becomes integrated into the host genome. It thus serves as a molecular marker for lentiviral VCN quantification via duplex qPCR. Plasmid standards (Lenti-genome-Copies) containing WPRE (vector-specific) and RNAseP (endogenous control) sequences were serially diluted in Yeast tRNA solution with NCG mouse matrix blanks to generate standard curves (R^2^ > 0.99). Experimental DNA samples were processed identically, with VCN determined by interpolating Ct values into the standard curve, enabling the normalization of vector copies to host genome equivalents. VCN was calculated using the formula: WPRE copy number×2/RNase P copy number.

### 2.6 Mononuclear cell isolation

The bone marrow cavities were subsequently flushed with DPBS utilizing a 1 mL syringe until the cavities appeared visibly devoid of residual marrow contents. The resultant cell suspension underwent filtration through a 70-µm cell strainer. A centrifugation process at 330 × g for 5 min at 4°C ensued to pellet the cells. The pelleted cells were subjected to treatment with red cell lysis buffer to eliminate red blood cells, followed by a washing step with DPBS. Subsequent experiments utilized the acquired bone marrow mononuclear cells.

Splenic mononuclear cell isolation involved the mechanical dissociation of spleens on a 70-µm strainer. The cell suspension was centrifuged at 330 *g* for 5 min at 4°C, after which red cell lysis and DPBS washing were performed to eradicate erythrocytes and debris.

Peripheral blood mononuclear cell isolation commenced with blood collection via retro-orbital puncture. Dual erythrocyte lysis cycles were implemented: blood samples were centrifuged at 400 *g* for 5 min at 4°C. The pellet was resuspended in red cell lysis buffer. This lysis procedure was repeated to ensure thorough removal of red blood cells. Ultimately, cells were washed with DPBS, and the resulting peripheral blood mononuclear cells were collected.

### 2.7 Flow cytometry analysis

Single-cell suspensions of peripheral blood, spleen, and bone marrow were prepared as described in method 2.6. Cells were adjusted to 1 × 10^6^ cells/mL in DPBS. For staining, 100 µL aliquots were incubated with 100 µL Zombie Aqua Fixable Viability Kit for 15 min at room temperature, protected from light. After DPBS washing (300 × g, 5 min), cells were resuspended in 100 µL DPBS and stained with pre-titrated antibody cocktails (Supplementary Table S5) for 30 min. Unbound antibodies were removed via two sequential DPBS washes (300 × g, 5 min). Final pellets were resuspended in 300 µL DPBS and stored at 4°C protected from light until acquisition. Representative gating schematics are provided in Supplementary Figures S1–4.

On D2, D57, and D92, femurs, tibias, and sternums were dissected to obtain bone marrow cells. Flow cytometry was used to assess the efficiency of erythroid maturation and differentiation by detecting hCD235a and hCD71 with the following gating strategy: mCD45^−^hCD45^−^hCD235a^+^CD71^-^ (mature erythrocytes), mCD45^−^hCD45^−^hCD71^+^CD235a^−^ (proerythroblasts), and mCD45^−^hCD45^−^hCD235a^+^CD71^+^ (intermediate erythroblasts).

On D2, D29, D57, and D92, peripheral blood, spleens, and bone marrow from femurs, tibias, and sternums were dissected. The analysis of myeloid and lymphoid cell differentiation cell populations was conducted as follows: T cells (hCD45^+^hCD3^+^), CD8^+^ T cells (hCD45^+^hCD3^+^hCD8^+^), CD4^+^ T cells (hCD45^+^hCD3^+^hCD4^+^), monocytes (hCD45^+^hCD13^+^), B cells (hCD45^+^hCD19^+^), and NK cells (hCD45^+^hCD56^+^). This analysis was conducted to evaluate myeloid and lymphoid cell differentiation. Additionally, hCD45 was measured in peripheral blood to calculate the peripheral blood chimerism rate.

All assays were performed using a DxFLEX cytometer, and data analysis was conducted using CytExpert for DxFLEX.

### 2.8 Colony forming cell (CFC) assay

Frozen cells were rapidly thawed at 37°C and diluted into 5 mL IMDM medium, followed by centrifugation at 500 *g* for 5 min. Viable cells were resuspended in IMDM, adjusted to 1.1 × 10^4^ cells/mL using trypan blue exclusion counting, and serially diluted in pre-warmed MethoCult H4435 Enriched Medium at a 1:10 ratio to achieve a final density of 1 × 10^3^ cells/mL. After vigorous vortex mixing (2–5 min) and 10-min deaeration at room temperature, 1 mL aliquots were plated in triplicate onto 6-well plates. Plates were gently tilted to ensure even distribution and incubated for 14 days at 37°C/5% CO_2_ under humidified conditions. Colony enumeration was performed manually using inverted microscopy, with morphological criteria distinguishing colony types: burst-forming unit erythroid (BFU-E), colony-forming unit granulocyte (CFU-G), macrophage (CFU-M), granulocyte-macrophage (CFU-GM), and multipotential granulocyte-erythrocyte-macrophage-megakaryocyte (CFU-GEMM) as defined by the manufacturer.

### 2.9 Cytokine analysis

Serum cytokines were quantified using the V-PLEX^®^ Proinflammatory Panel 1 Kit on a QuickPlex SQ 120 platform. Samples (2-fold diluted in Diluent 41) underwent 2 h incubation at 25°C with orbital shaking (600 rpm). Post-washing (PBS-T, 3×), SULFO-TAG-conjugated detection antibodies were added (1 h incubation), followed by Read Buffer T application and electrochemiluminescence signal measurement. Analytes included IFN-γ (0.37–1,500 pg/mL), IL-2 (0.37–1,530 pg/mL), IL-4 (0.06–258 pg/mL), IL-6 (0.18–720 pg/mL), IL-10 (0.09–384 pg/mL), and TNF-α (0.09–362 pg/mL). Measurements below detection thresholds were excluded per manufacturer specifications.

### 2.10 Hematoxylin and eosin (H&E) staining

Tissue sections (4 µm) were deparaffinized in xylene, rehydrated through graded ethanol, and stained with Mayer’s hematoxylin (10 min). After differentiation (1% acid ethanol, 1 s) and bluing (tap water, 7 min), cytoplasmic counterstaining was performed with eosin Y (5 min). Sections were dehydrated, cleared in xylene, and mounted with neutral resin for brightfield microscopy.

### 2.11 Toxicokinetic and distribution analysis

Mice were euthanized on D2, D29, D57, and D92 following the injection. Blood (with EDTA anticoagulant) and tissues, including bone marrow, spinal cord, brain, lungs, heart, liver, spleen, kidneys, skeletal muscle, joints, stomach, duodenum, jejunum, colon, epididymis, ovaries, or whole blood, were collected from each mouse. Samples were temporarily stored on ice and quickly transferred to conditions below −70°C for tissue distribution analysis. Genomic DNA was extracted using the MagaBio Plus Universal Genomic DNA Purification Kit II. Quantitative PCR was employed to analyze the WPRE gene DNA in mouse tissues to determine the *in vivo* distribution of BD211. The PCR parameters were set to 37°C for 2 min, 95°C for 5 min, followed by 45 cycles of 95°C for 15 s and 60°C for 40 s. A standard curve was generated by linear regression of the WPRE plasmid Ct and concentration log values. The actual sample Ct values were substituted into the standard curve to calculate the WPRE DNA content in whole blood and tissue samples. The primer sequence information is detailed in Supplementary Table S4.

### 2.12 Quantification of WT/GAPDH and T87Q/GAPDH mRNA

Total RNA was extracted from tissues using the MagaBio Plus Total RNA Purification Kit II (BSC69L1E/BSC69M1E, BioFlux) and from whole blood and bone marrow using the Whole Blood Total RNA Kit (5201050, Hangzhou Xingjing Biotech Co., Ltd.). Quantitative PCR was used to analyze the human β^A^-globin, wild-type β^A^-globin, and GAPDH mRNA in mouse tissues. The parameters were set to 95°C 15 min; 95°C 10 s, 60°C 20 s, 72°C 20 s, and 40 cycles. The Ct values for the samples were obtained using the instrument software. The relative expression levels were calculated as follows:

WT/GAPDH (wild-type β^A^-globin/GAPDH) = Ct_WT_/Ct _GAPDH_×100%;

T87Q/GAPDH (β^A−T87Q^-globin/GAPDH) = Ct_T87Q_/Ct _GAPDH_×100%

### 2.13 Oncogenicity assessment

All preserved tissues and organs from the vehicle control, mock control, and high-dose BD211 groups underwent microscopic examination for proliferative or neoplastic changes using H&E staining. Should tumors be detected in high-dose animals, corresponding organs from low-dose groups would have been analyzed.

### 2.14 Statistical analysis

Statistical analyses were performed using SPSS Statistics for Windows (v.23.0, IBM Corp., Armonk, N.Y., United States). Measurement data, including body weight, food intake, hematological indicators, serum biochemical indicators, and organ weight and coefficient, were presented as mean ± standard deviation (x ± SD). One-way analyses of variance (ANOVAs) were used to assess significance. If a statistically significant difference was found among the groups, further pairwise comparisons were conducted. Levene’s test was used to determine the homogeneity of variance: if groups were homoscedastic, the least significant difference method was applied, and if heteroscedastic, the Games-Howell test was used for pairwise comparisons. Count data, such as behavioral and pathological incidences, were described as fractions, and non-parametric tests (Kruskal–Wallis test) were employed for significance analysis. When a statistically significant difference was observed among the overall groups, differences between pairwise groups were compared. The P-value of <0.05 was considered statistically significant.

## 3 Results

### 3.1 *In vitro* differentiation, β^A−T87Q^-globin expression, and VCN of BD211

**FIGURE 2:**
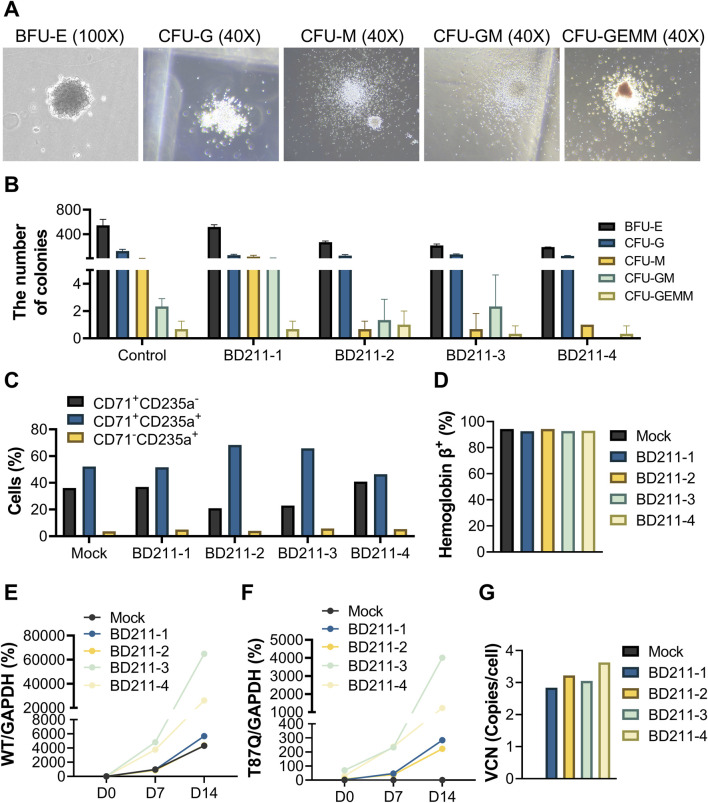
Erythroid differentiation of BD211 *in vitro* and VCN. **(A).** Typical representative micrograph of five colonies after 14 days of *in vitro* differentiation of BD211. **(B).** Quantification of colony subtypes from **(A)**, determined by manual counting of triplicate wells using morphological criteria. **(C).** Proportion of red blood cells after 14 days of culture of mock cells and BD211 *in vitro*. CD71^+^CD235a^−^: Proerythroblasts; CD71^+^CD235a^+^: Basophilic, Polychromatophilic & Orthochromatic Erythroblasts; CD71^-^CD235a^+^: Reticulocytes & Mature Red Blood Cells. **(D).** Percentage of hemoglobin β^+^cells cultured *in vitro* after 14 days **(E,F).** The mRNA expression levels of β^A^-globin and β^A−T87Q^-globin on days 0, 7, and 14. Wild-type β^A^-globin/GAPDH (WT/GAPDH) = Ct_WT_/Ct _GAPDH_×100%; β^A−T87Q^-globin/GAPDH (T87Q/GAPDH) = Ct _T87Q_/Ct _GAPDH_×100% **(G).** The VCN value of mock cells and BD211 cultured *in vitro* after 14 days. VCN = WPRE/(Rnase P/2). BFU-E: burst-forming unit erythroid; CFU-G: colony-forming unit granulocyte; CFU-M: colony-forming unit macrophage; CFU-GM: colony-forming unit granulocyte-macrophage; and CFU-GEMM: colony-forming unit multipotential granulocyte-erythrocyte-macrophage-megakaryocyte; VCN: Vector Copy Number.

The colony-forming cell assay is an *in vitro* method used to assess the proliferation and differentiation capabilities of hematopoietic stem cells (Kronstein-Wiedemann and Tonn, 2019). Both the mock control and test groups were cultured using semi-solid culture media for 14 days and successfully differentiated into 5 colony types: erythrocytic burst-forming unit, granulocyte colony-forming unit, macrophage colony-forming unit, granulocyte-macrophage colony-forming units, and granulocyte, erythrocyte, macrophage, and megakaryocyte colony-forming units (Figures 2A,B).

During the directed differentiation of CD34^+^ hematopoietic stem cells into erythrocytes, the expression of CD71 gradually decreased, while that of CD235a increased. Early erythroid precursors (proerythroblasts) primarily exhibited a CD71^+^CD235a^−^, intermediate erythroid precursors (basophilic, polychromatophilic, and orthochromatic erythroblasts) exhibited a CD71^+^CD235a^+^ and mature erythrocytes (reticulocytes and mature red blood cells) predominantly displayed CD71^-^CD235a^+^ markers. Both mock control cells and four batches of test cells successfully differentiated into erythroid cells *in vitro* (Figure 2C).

Hemoglobin, the primary component of erythrocytes, is composed of three proteins, with hemoglobin A (HbA) being the most abundant, accounting for over 95% of total hemoglobin in the human body. Flow cytometry analysis revealed that both mock control cells and four batches of test cells successfully detected HbA-positive cells *in vitro*, with positivity exceeding 90% (Figure 2D).

The relative expression levels of WT/GAPDH in mock control cells and four batches of BD211 cells significantly increased with prolonged *in vitro* differentiation time. Similarly, the relative expression levels of T87Q/GAPDH in the four batches of BD211 cells also significantly increased. T87Q/GAPDH expression was not detected in mock control cells throughout the experiment (Figures 2E,F).

The mock control cells did not exhibit positivity for the BD211 vector, whereas all four batches of test groups showed BD211 vector positivity, with VCN ranging from 2.84 to 3.63 (Figure 2G). The VCN values for all four batches of test groups were within the safety range of 1–5 copies/cell in accordance with relevant guidelines (U. S. Food and Drug, 2024).

These results demonstrate that BD211 successfully differentiated into various stages of human erythroid cells *in vitro* and expressed β^A−T87Q^-globin during the directed differentiation process.

### 3.2 Basic physiological and behavioral monitoring in animals

**FIGURE 3:**
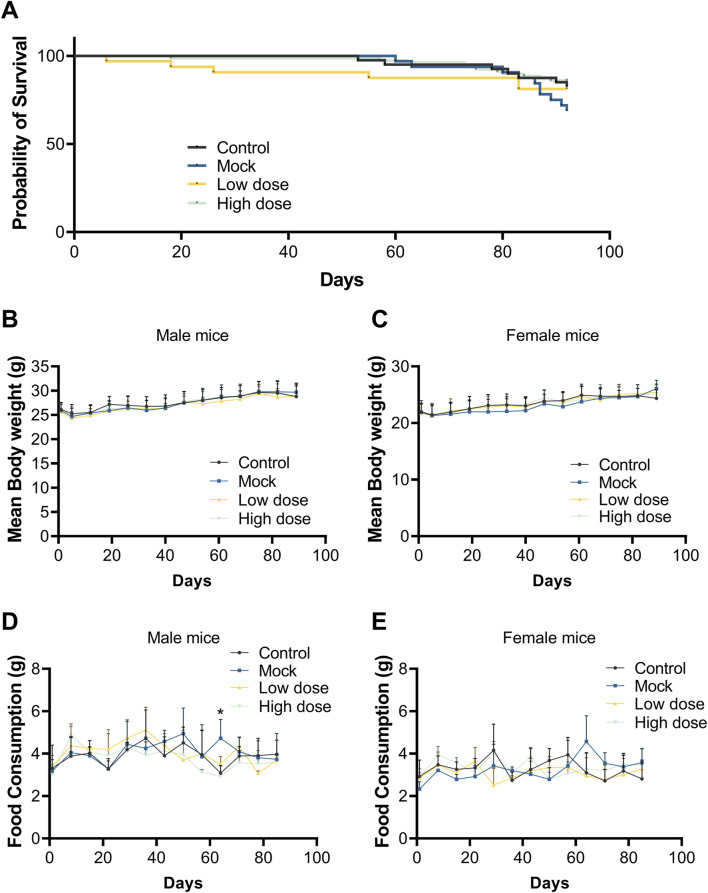
Survival curve, body weight, and food consumption after a single dose of BD211. **(A).** Survival curve of NCG-X mice from D0 to D92. Body weight of male **(B)** and female **(C)** mice from D0 to D89. Food consumption of male **(D)** and female **(E)** mice from D0 to D85. Data are expressed as the mean ± SD and were analyzed using one-way ANOVAs.

During the trial period, mortality or near-death events occurred in all groups, with proportions of 17.50%, 19.23%, 11.54%, and 14.10%, respectively. No dose-dependent relationship was observed, and these events were not attributed to the test substance (Figure 3A).

Throughout the study, there were no significant differences in body weight gain between the BD211 low- and high-dose groups and the solvent control group for both male and female mice. Body weight increased over time in all groups (Figures 3B,C). Food consumption in BD211 groups fluctuated within normal ranges, with no significant changes noted. A transient increase in food consumption was observed in male mice of the mock control group, which was not considered toxicologically significant (Figures 3D,E).

### 3.3 Hematology and biochemical assessment

At the end of the 13-week dosing period (D92), the hematological parameters of the mock control and BD211 groups, including red blood cell indices (RBC, HGB, HCT, MCHC, and RET), white blood cell indices (WBC, NEUT, LYMPH, MONO), and platelets (PLT), showed a decrease in absolute values compared to the solvent control group, exhibiting a dose-dependent relationship with the human CD34^+^ hematopoietic stem cell dose (Table 1). No biochemically significant changes were observed in the BD211 low- and high-dose groups (Supplementary Table S1).

**TABLE 1:** Hematology analysis of NCG-X mice treated with BD211 on D92. Blood samples collected in EDTA-K2 were analyzed within 8 h at room temperature using the Automatic Hematology Analyzer XN-1000V.

Parameter	Male mice				Female mice			
	Control	Mock	Low dose	High dose	Control	Mock	Low dose	High dose
Number of animals	9	11	12	11	8	4	9	8
RBC (10^12^/L)	5.520 ± 0.303	3.803 ± 1.134**	5.357 ± 0.316^##^	4.937 ± 0.250***^#^	5.299 ± 0.779	3.588 ± 0.685***	4.972 ± 0.290^###^	4.483 ± 0.420*^#^
HGB (g/L)	102.9 ± 4.9	72.8 ± 20.0**	101.0 ± 5.0^###^	95.3 ± 3.8**^##^	100.1 ± 15.0	70.3 ± 12.8***	96.6 ± 6.1^###^	87.9 ± 7.1^##^
HCT (%)	33.46 ± 1.69	24.55 ± 5.93**	32.88 ± 1.41^##^	31.35 ± 1.26**^##^	32.65 ± 4.68	24.15 ± 3.57***	31.91 ± 1.99^###^	28.90 ± 2.42^#^
MCV (fL)	60.63 ± 0.84	65.44 ± 3.63**	61.47 ± 1.72^##^	63.55 ± 1.44***	61.66 ± 0.82	67.83 ± 3.46**	64.17 ± 1.02***^#^	64.53 ± 1.21***
MCH (Pg)	18.64 ± 0.32	19.25 ± 0.63*	18.86 ± 0.47	19.31 ± 0.48*	18.88 ± 0.50	19.63 ± 0.44*	19.40 ± 0.33	19.63 ± 0.49**
MCHC (g/L)	307.4 ± 2.8	294.5 ± 9.9**	307.2 ± 3.3^##^	303.8 ± 2.9*^#^	306.3 ± 7.6	289.8 ± 13.7	302.6 ± 4.5	304.0 ± 5.6
#RETIC (10^9^/L)	296.99 ± 39.99	235.12 ± 61.72*	309.48 ± 41.35^##^	289.78 ± 50.59^#^	317.31 ± 32.81	401.88 ± 122.44	330.76 ± 82.77	297.43 ± 83.70
%RETIC (%)	5.369 ± 0.535	6.368 ± 1.306	5.779 ± 0.689	5.848 ± 0.843	6.089 ± 0.936	11.253 ± 2.926***	6.656 ± 1.586^##^	6.715 ± 2.159^##^
WBC (10^9^/L)	0.988 ± 0.686	0.351 ± 0.142**	0.753 ± 0.564^##^	0.424 ± 0.192*	0.479 ± 0.134	0.323 ± 0.074	0.509 ± 0.308	0.738 ± 0.994
#NEUT (10^9^/L)	0.584 ± 0.444	0.192 ± 0.087**	0.497 ± 0.384^##^	0.249 ± 0.110*	0.273 ± 0.089	0.200 ± 0.056	0.290 ± 0.190	0.503 ± 0.815
%NEUT (%)	59.09 ± 10.50	54.17 ± 7.60	65.37 ± 6.23^##^	59.11 ± 7.07	57.55 ± 11.90	61.55 ± 4.2	56.71 ± 14.16	58.29 ± 11.71
#LYMPH (10^9^/L)	0.248 ± 0.182	0.103 ± 0.037*	0.158 ± 0.125	0.105 ± 0.052*	0.140 ± 0.060	0.078 ± 0.021	0.131 ± 0.090	0.138 ± 0.094
%LYMPH (%)	24.41 ± 5.85	30.08 ± 7.15	20.78 ± 4.51^##^	25.44 ± 6.90	28.70 ± 9.39	24.03 ± 4.27	25.53 ± 10.41	25.79 ± 10.49
#MONO (10^9^/L)	0.153 ± 0.092	0.051 ± 0.021**	0.098 ± 0.062	0.068 ± 0.040*	0.066 ± 0.026	0.045 ± 0.006	0.086 ± 0.052	0.084 ± 0.058
%MONO (%)	16.28 ± 5.57	14.60 ± 1.81	13.85 ± 2.88	15.29 ± 3.43	13.75 ± 3.66	14.43 ± 3.47	17.27 ± 4.34	15.50 ± 4.25
#EOS (10^9^/L)	0.002 ± 0.004	0.005 ± 0.015	0 ± 0	0.001 ± 0.003	0 ± 0	0 ± 0	0.002 ± 0.007	0.013 ± 0.035
%EOS (%)	0.22 ± 0.45	1.15 ± 2.78	0 ± 0	0.16 ± 0.54	0 ± 0	0 ± 0	0.49 ± 1.47	0.39 ± 1.10
#BASO (10^9^/L)	0 ± 0	0 ± 0	0 ± 0	0 ± 0	0 ± 0	0 ± 0	0 ± 0	0.001 ± 0.004
%BASO (%)	0 ± 0	0 ± 0	0 ± 0	0 ± 0	0 ± 0	0 ± 0	0 ± 0	0.04 ± 0.11
PLT (10^9^/L)	1,363.4 ± 155.3	938.0 ± 230.0***	1,348.8 ± 274.3^###^	1,179.8 ± 128.4^#^	1,062.5 ± 402.3	786.5 ± 65.6	1,022.6 ± 90.8^##^	939.8 ± 111.4^#^

Note: The data were expressed as mean ± SD. “*”,“**” or,“***” indicates a statistically significant difference at *p* < 0.05, *p* < 0.01 or *p* < 0.001 when compared to the control group; “#”,“##” or,“###” indicates a statistically significant difference at *p* < 0.05, *p* < 0.01 or *p* < 0.001 when compared to the mock group; Abbreviation: RBC, red blood cell count; HGB, hemoglobin concentration; HCT, hematocrit; MCV, mean cell volume; MCH, mean cell hemoglobin; MCHC, mean cell hemoglobin concentration; RETIC, reticulocyte; WBC, white blood cell; NEUT, neutrophils; LYMPH, lymphocytes; MONO, monocytes; EOS, eosinophils; BASO, basophils; PLT, platelets.

### 3.4 Therapeutic efficacy

From D29 onwards, the percentage of hCD3^+^, hCD13^+^, hCD19^+^, hCD34^+^, and hCD56^+^ in the bone marrow of both the mock control and BD211 groups increased in a dose-dependent manner, reaching the highest level by D92 (Table 2). Similarly, starting from D29, the percentage of hCD3^+^, hCD13^+^, and hCD19^+^ in the spleen of the mock control and BD211 groups increased in a dose-dependent manner, reaching the highest level by D92. The percentages of other indicators (hCD4^+^, hCD8^+^, hCD56^+^) did not show significant changes compared to their D2 levels (Table 3).

**TABLE 2:** Myeloid and lymphoid cell differentiation in the bone marrow of NCG-X mice treated with BD211 on D2, D29, D57, and D92, as analyzed by flow cytometry**.**

Parameter		Female mice				Male mice			
		Control	Mock	Low dose	High dose	Control	Mock	Low dose	High dose
hCD45^+^hCD3^+^ in hCD45^+^/mCD45^+^ (%)	D2	0.003 ± 0.005	0 ± 0	0 ± 0	0.153 ± 0.072*^#^	0 ± 0	0.003 ± 0.006	0.023 ± 0.021	0.077 ± 0.080
	D29	0.003 ± 0.005	3.153 ± 1.092*	0.760 ± 0.421*^#^	2.155 ± 0.896*	0 ± 0	1.913 ± 1.187*	0.110	0.772 ± 0.523*
	D57	—	5.843 ± 1.698	2.668 ± 0.445^#^	4.439 ± 1.695	—	4.363 ± 1.768	1.645 ± 1.082	3.549 ± 2.065
	D92	—	7.348 ± 1.629	4.278 ± 1.096^#^	4.134 ± 1.324^#^	—	5.953 ± 2.597	1.150 ± 0.290	5.562 ± 3.768
hCD45^+^hCD34^+^ in hCD45^+^/mCD45^+^ (%)	D2	0 ± 0	0.033 ± 0.006*	0.010 ± 0.010	0.102 ± 0.062*	0.003 ± 0.005	0.013 ± 0.006	0.033 ± 0.035	0.058 ± 0.062
	D29	0.005 ± 0.010	13.247 ± 5.610*	4.127 ± 2.134*	10.342 ± 4.119*	0.003 ± 0.005	5.983 ± 3.985*	0.470	3.183 ± 1.691*
	D57		23.108 ± 2.932	13.660 ± 2.316^#^	19.796 ± 4.029		16.883 ± 3.954	5.933 ± 4.311^#^	12.990 ± 6.579
	D92		24.018 ± 7.064	22.425 ± 2.086	20.182 ± 3.358		24.407 ± 5.124	7.837 ± 3.650^#^	16.224 ± 3.834^#^
hCD45^+^hCD13^+^ in hCD45^+^/mCD45^+^ (%)	D2	0 ± 0	0.033 ± 0.006*	0.010 ± 0.010^#^	0.182 ± 0.089*^#^	0 ± 0	0.013 ± 0.006	0.063 ± 0.047	0.112 ± 0.130
	D29	0.005 ± 0.010	8.830 ± 3.717*	2.087 ± 0.880*^#^	4.403 ± 1.653*^#^	0 ± 0	4.090 ± 2.869*	0.320	1.238 ± 0.479*^#^
	D57	—	23.595 ± 3.188	6.145 ± 1.241^#^	16.164 ± 7.083	—	13.643 ± 6.386	2.500 ± 1.115^#^	5.746 ± 3.250^#^
	D92	—	28.055 ± 6.003	18.420 ± 1.660^#^	15.544 ± 8.343^#^	—	25.107 ± 12.945	2.837 ± 0.448^#^	13.766 ± 6.263
hCD45^+^hCD19^+^ in hCD45^+^/mCD45^+^ (%)	D2	0.003 ± 0.005	0.003 ± 0.006	0.033 ± 0.058	0.245 ± 0.109*^#^	0.008 ± 0.005	0.003 ± 0.006	0.230 ± 0.215	0.305 ± 0.245
	D29	0.003 ± 0.005	8.770 ± 4.465*	3.830 ± 1.537*	12.992 ± 6.008*	0.003 ± 0.005	3.670 ± 2.600*	0.420	3.515 ± 1.676*
	D57	—	48.148 ± 6.956	31.443 ± 7.352^#^	46.027 ± 7.721		35.840 ± 5.665	12.115 ± 10.960	26.793 ± 13.707
	D92	—	46.065 ± 4.312	56.003 ± 4.712	51.752 ± 9.378		46.690 ± 9.047	24.337 ± 15.415	43.944 ± 11.964
hCD45^+^hCD56^+^ in hCD45^+^/mCD45^+^ (%)	D2	0 ± 0	0.003 ± 0.006	0.007 ± 0.012	0.217 ± 0.158*^#^	0.008 ± 0.005	0.003 ± 0.006	0.077 ± 0.068	0.145 ± 0.182
	D29	0.003 ± 0.005	0.720 ± 0.139*	0.273 ± 0.126*^#^	0.633 ± 0.203*	0 ± 0	0.543 ± 0.281*	0.030	0.268 ± 0.133*
	D57		1.728 ± 0.568	0.653 ± 0.171^#^	1.076 ± 0.211^#^		1.505 ± 0.702	0.478 ± 0.438^#^	0.857 ± 0.402
	D92		1.405 ± 0.422	0.738 ± 0.139^#^	1.036 ± 0.298		1.933 ± 1.099	0.537 ± 0.234	1.764 ± 1.675

Note: The data were expressed as mean ± SD. “*”or“**” indicates a statistically significant difference at *p* < 0.05 and *p* < 0.01 when compared to the control group; “#”,“##” or,“###” indicates a statistically significant difference at *p* < 0.05, *p* < 0.01 or *p* < 0.001 when compared to the mock group. The percentages indicate the proportion of cells expressing the specified antigens. For gating strategy details, refer to the Methods section. h: human; m: mouse; hCD45/mCD45^+^ indicates the presence of either human CD45^+^ cells or mouse CD45^+^ cells.

**TABLE 3:** Myeloid and lymphoid cell differentiation in the spleen of NCG-X mice treated with BD211 on D2, D29, D57, and D92, as analyzed by flow cytometry**.**

Parameter		Female mice				Male mice			
		Control	Mock	Low dose	High dose	Control	Mock	Low dose	High dose
hCD45^+^hCD3^+^ in hCD45^+^/mCD45^+^ (%)	D2	0.015 ± 0.006	0.140 ± 0.036*	0.053 ± 0.055	0.025 ± 0.021^#^	0.025 ± 0.013	0.127 ± 0.070*	0.003 ± 0.006*^#^	0.027 ± 0.033^#^
	D29	0 ± 0	0.230 ± 0.159	0.053 ± 0.055	0.143 ± 0.119	0 ± 0	0.137 ± 0.142	0.020	0.123 ± 0.102
	D57	—	0.340 ± 0.096	0.153 ± 0.093	0.301 ± 0.119	—	0.688 ± 0.198	0.095 ± 0.098^#^	0.250 ± 0.128^#^
	D92	—	1.015 ± 0.205	0.945 ± 0.747	0.920 ± 0.593	—	0.987 ± 0.127	0.163 ± 0.100^#^	1.106 ± 0.821
hCD45^+^hCD3^+^hCD8^+^ in hCD45^+^/mCD45^+^ (%)	D2	0.003 ± 0.005	0 ± 0	0.007 ± 0.006	0.000 ± 0.000	0.008 ± 0.005	0.003 ± 0.006	0.003 ± 0.006	0.002 ± 0.004
	D29	0 ± 0	0 ± 0	0 ± 0	0 ± 0	0 ± 0	0 ± 0	0 ± 0	0 ± 0
	D57	—	0 ± 0	0.003 ± 0.005	0.001 ± 0.004	—	0 ± 0	0 ± 0	0.001 ± 0.004
	D92	—	0.008 ± 0.005	0.018 ± 0.010	0.004 ± 0.005	—	0.010 ± 0.000	0 ± 0	0.006 ± 0.009
hCD45^+^hCD3^+^hCD4^+^ in hCD45^+^/mCD45^+^ (%)	D2	0.008 ± 0.005	0 ± 0	0.007 ± 0.012	0.002 ± 0.004	0 ± 0	0.003 ± 0.006	0 ± 0	0 ± 0
	D29	0 ± 0	0 ± 0	0 ± 0	0 ± 0	0 ± 0	0	0 ± 0	0 ± 0
	D57	—	0 ± 0	0 ± 0	0 ± 0	—	0.003 ± 0.005	0 ± 0	0 ± 0
	D92	—	0.013 ± 0.013	0.015 ± 0.017	0.018 ± 0.016	—	0.007 ± 0.006	0 ± 0	0.012 ± 0.011
hCD45^+^hCD13^+^ in hCD45^+^/mCD45^+^ (%)	D2	0.010 ± 0.008	0.597 ± 0.155*	0.113 ± 0.023*^#^	0.367 ± 0.159*	0.018 ± 0.013	0.450 ± 0.131*	0.130 ± 0.020*^#^	0.352 ± 0.140*
	D29	0 ± 0	0.790 ± 0.762*	0.157 ± 0.139*	0.440 ± 0.229*	0	0.397 ± 0.318*	0.030	0.237 ± 0.170*
	D57	—	1.268 ± 0.342	0.370 ± 0.227^#^	1.153 ± 0.699	—	2.090 ± 0.652	0.245 ± 0.259^#^	0.654 ± 0.344^#^
	D92	—	4.163 ± 0.598	3.348 ± 3.166	3.038 ± 2.018	—	3.747 ± 0.129	0.440 ± 0.304^#^	4.478 ± 2.903
hCD45^+^hCD19^+^ in hCD45^+^/mCD45^+^ (%)	D2	0.010 ± 0.000	0.037 ± 0.015	0.050 ± 0.056	0.035 ± 0.029	0.028 ± 0.013	0.040 ± 0.020	0.010 ± 0.017	0.025 ± 0.029
	D29	0 ± 0	0.517 ± 0.497*	0.217 ± 0.204*	0.420 ± 0.329*	0 ± 0	0.160 ± 0.118	0.030	0.205 ± 0.171
	D57	—	16.540 ± 1.889	3.935 ± 2.035^#^	11.033 ± 4.728		16.288 ± 2.760	2.128 ± 1.924^#^	4.241 ± 2.219^#^
	D92	—	39.985 ± 5.676	22.355 ± 14.315	27.784 ± 17.623		30.257 ± 5.020	3.277 ± 2.173^#^	22.364 ± 14.396
hCD45^+^hCD56^+^ in hCD45^+^/mCD45^+^ (%)	D2	0.010 ± 0.008	0.080 ± 0.026	0.057 ± 0.074	0.020 ± 0.021	0.028 ± 0.013	0.053 ± 0.023	0.003 ± 0.006*^#^	0.015 ± 0.023
	D29	0 ± 0	0.167 ± 0.159*	0.067 ± 0.045*	0.092 ± 0.080*	0 ± 0	0.077 ± 0.067	0.020	0.077 ± 0.066
	D57	—	0.585 ± 0.159	0.210 ± 0.154^#^	0.476 ± 0.131		0.795 ± 0.161	0.115 ± 0.138^#^	0.280 ± 0.154^#^
	D92	—	1.140 ± 0.143	0.798 ± 0.382	0.890 ± 0.524		1.157 ± 0.032	0.193 ± 0.116	1.192 ± 0.730

Note: The data were expressed as mean ± SD. “*“or“**” indicates a statistically significant difference at *p* < 0.05 and *p* < 0.01 when compared to the control group; “#“,“##” or,“###” indicates a statistically significant difference at *p* < 0.05, *p* < 0.01 or *p* < 0.001 when compared to the mock group. The percentages indicate the proportion of cells expressing the specified antigens. For gating strategy details, refer to the Methods section. h: human; m: mouse; hCD45/mCD45^+^ indicates the presence of either human CD45^+^ cells or mouse CD45^+^ cells.

From D2 to D92, the levels of hCD3^+^, hCD4^+^, hCD8^+^, hCD4^+^/hCD8^+^, hCD56^+^, hCD13^+^, and hCD19^+^ in peripheral blood of the mock control and BD211 groups remained very low level, with no significant increase. This was likely due to the residual immune function of NCG-X mice, as suggested by background information from the animal supplier (unpublished), which indicated that monocytes or macrophages in the tissues could phagocytose and clear the xenogeneic human terminal blood cells released into the peripheral blood after the reconstruction and differentiation of human CD34^+^ hematopoietic stem cells (Supplementary Table S2).

**FIGURE 4:**
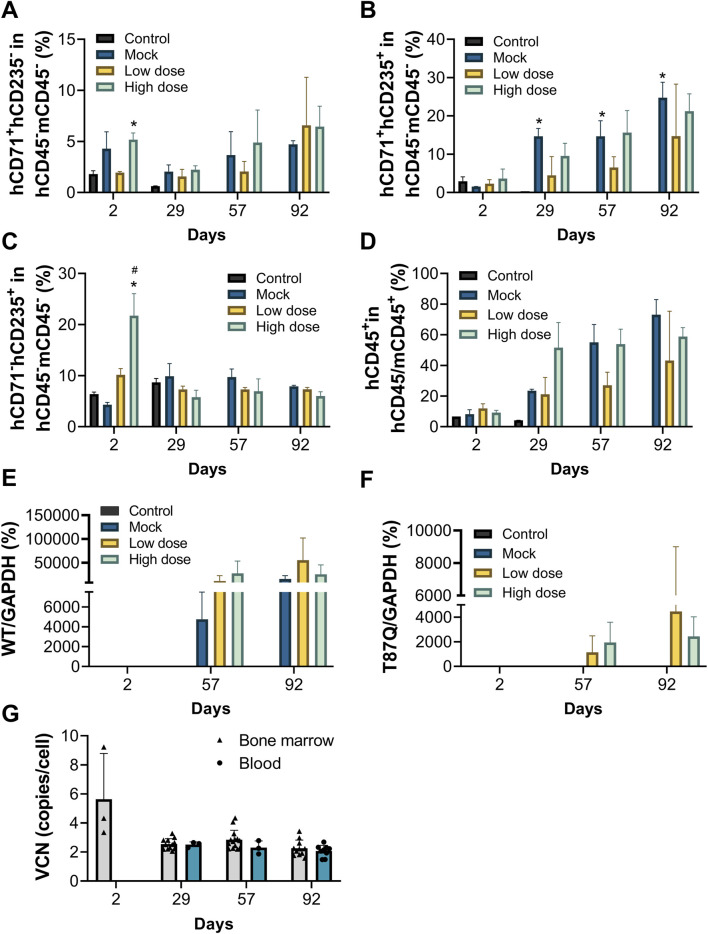
Erythroid differentiation *in vivo*, peripheral blood chimerism rate and VCN of NCG-X mice after a single administration of BD211. (**A–C).** The Proportion of red blood cells in the bone marrow of NCG-X mice on D2, D29, D57, and D92 was analyzed by flow cytometry. h: human; m: mouse. *p < 0.05 (one-way ANOVA compared with the Control group); #p < 0.05 (one-way ANOVA compared with the Mock group). **(D).** peripheral blood chimerism rate in blood of NCG-X mice on D2, D29, D57, and D92 analyzed by flow cytometry. hCD45/mCD45^+^ indicates human CD45^+^ cells and/or mouse CD45^+^ cells. **(E,F).** mRNA expression levels of β^A^-globin and β^A−T87Q^-globin in the bone marrow of NCG-X mice on D2, D29, D57, and D92. **(G).** The VCN value of BD211 cultured in bone marrow and blood of NCG-X mice on D2, D29, D57, and D92.

Both the mock control and BD211 groups successfully differentiated into erythroid cells in the bone marrow, with a significant increase in the percentage of hCD71^+^hCD235a^+^ cells, indicating a high proportion of immature erythrocytes, consistent with *in vitro* differentiation results (Figures 4A–C).

Starting from D29, the percentage of hCD45^+^ cells and their proportion in peripheral blood increased in a dose-dependent manner for both the mock control and BD211 groups, reaching the highest level by D92. On D92, the hCD45^+^ chimerism rates for the mock, BD211 low-dose, and high-dose groups were 73.10%, 43.24%, and 58.85%, respectively (Figure 4D).

**FIGURE 5:**
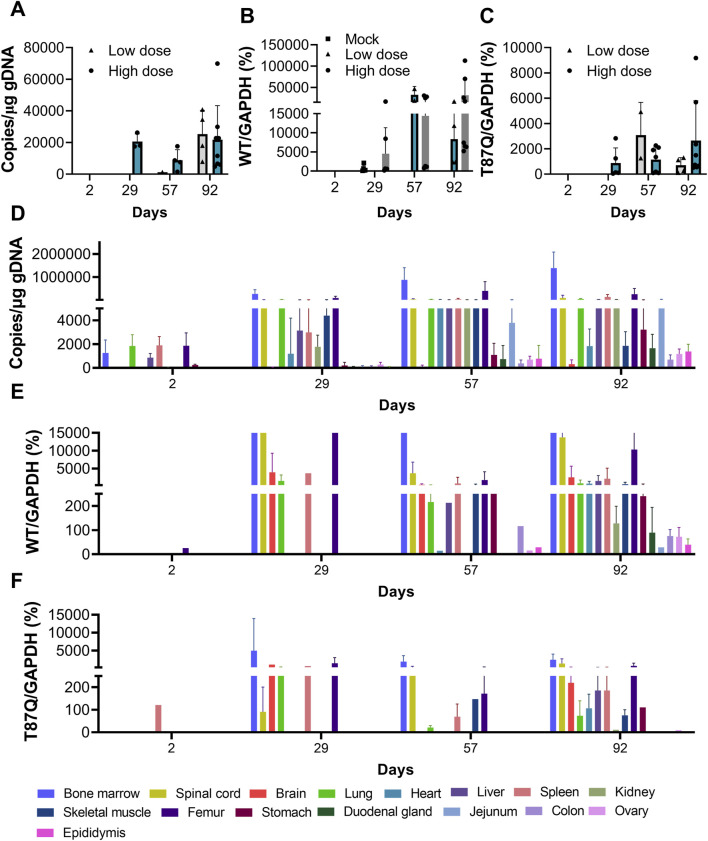
Toxicokinetics and distribution of BD211 in NCG-X mice. (**A).** Concentration of BD211 in the blood of NCG-X mice on D2, D29, D57, and D92. (**B,C).** The mRNA expression levels of β^A^-globin and β^A−T87Q^-globin in the blood of NCG-X mice on D2, D29, D57, and D92. **(D).** Concentration of BD211 in tissues of NCG-X mice on D2, D29, D57, and D92. **(E,F).** The mRNA expression levels of β^A^-globin and β^A−T87Q^-globin in tissues of NCG-X mice on D2, D29, D57, and D92.

On D29, occasional expression of β-globin mRNA and β^A−T87Q^-globin mRNA was observed in the bone marrow in the BD211 high-dose group (Figures 5E,F). By D57 and D92, widespread expression of β-globin mRNA was observed in the mock group, whereas β-globin mRNA and β^A−T87Q^-globin mRNA in BD211 groups were detected in the bone marrow (Figures 4E,F).

In the high-dose group, WPRE was detected in the bone marrow of only a few mice (3/12) on D2, with a VCN value of 5.63. From D29 onwards, WPRE was consistently detected in the bone marrow and blood of all mice, with VCN values ranging from 2.07 to 2.84. No significant inter-sample or gender differences in VCN values were observed, and VCN values remained relatively stable throughout the study (Figure 4G).

During the study, human-specific cytokine levels of IL-2, IL-4, IL-6, IL-10, TNF-α, and IFN-γ were mostly below the limit of quantification for all groups at each testing point, failing to meet the requirements for statistical analysis (data not shown).

### 3.5 Toxicokinetics and distribution

From D2 to D92, the detection rate and genomic concentration of BD211 (representing BD211 and its differentiated nucleated cells in toxicokinetic and tissue distribution studies, with the WPRE gene as the detection marker) increased with the extension of observation time. In the early stages of administration (D2 to D57), the concentration of BD211 in the blood of high-dose animals was significantly higher than that of the low-dose group. However, as time progressed, by D92, the BD211 concentrations in the blood of both groups tended to converge (Figure 5A). The expression trends of β^A−T87Q^-globin and β-globin mRNA were essentially consistent with the distribution trend of BD211. Over time, the concentration and detection rate of BD211 in the blood and the detection rate and concentration levels of β^A−T87Q^-globin and β-globin mRNA exhibited a clear increasing trend within a certain range (Figures 5B,C).

On D2, BD211 was predominantly detected in the bone marrow, lungs, liver, spleen, femur, and stomach, with concentrations ranging from 1.06 × 10^2^ to 4.71 × 10^3^ copies/μg gDNA. Over time, the concentration and distribution range of BD211 gradually increased. By D29, it was distributed in multiple organs, with the highest concentration in the bone marrow, followed by the elbow joint, while concentrations in other tissues were relatively lower. The overall detection concentration range was 1.12 × 10^2^ to 5.34 × 10^5^ copies/μg gDNA, with specific ranges of 2.07 × 10^4^ to 5.34 × 10^5^ copies/μg gDNA in the bone marrow and 3.54 × 10^3^ to 2.12 × 10^5^ copies/μg gDNA in the elbow joint. The distribution trend of BD211 in various tissues and organs at D57 and D92 was consistent with that of D29. The concentration growth trend from D57 to D92 was not significant, suggesting a possible steady state (Figure 5D).

The changes in β^A−T87Q^-globin mRNA and β-globin mRNA were largely consistent. They were primarily detected in tissues, such as the lungs, elbow joint, bone marrow, and spinal cord. Over time, the range of detected tissues gradually increased until almost all tissues were involved (Figures 5E,F).

### 3.6 Anatomical and pathological safety evaluation

At D92, gross pathological examination of animals in the BD211 groups revealed no gross pathological changes related to the test substance nor any proliferative or tumorigenic changes associated with BD211.

**FIGURE 6:**
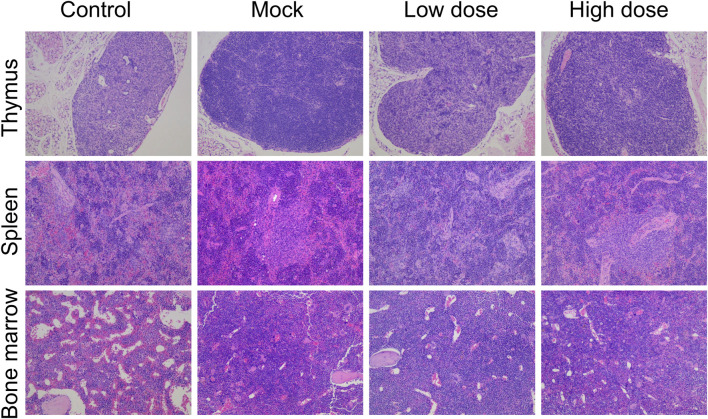
H&E staining of the thymus, spleen, and bone marrow of NCG-X mice after a single administration of BD211.

The spleen weight, as well as the organ-to-body and organ-to-brain coefficients, were significantly elevated in male animals of the BD211 high-dose and mock control groups (Table 4). In both male and female mice of the BD211 high-dose and mock groups, an increase in the number of bone marrow (sternum) cells, thymic lymphocytes, and cells surrounding the splenic arteries was observed. Additionally, extramedullary hematopoiesis was observed in male animals. In the BD211 low-dose group, both male and female animals exhibited increased bone marrow (sternum) cells, and extramedullary hematopoiesis was noted in male animals (Figure 6). These histopathological changes were due to the engraftment, reconstitution, and differentiation of human CD34^+^ hematopoietic stem cells in NCG-X mice rather than specific changes induced by the test substance BD211.

**TABLE 4:** Organ weight and coefficient of NCG-X mice treated with BD211 on D92.

Parameter		Male mice				Female mice			
		Control	Mock	Low dose	High dose	Control	Mock	Low dose	High dose
Number of animals		9	11	12	11	8	4	9	8
Heart	Weight (g)	0.125 ± 0.012	0.138 ± 0.009*	0.127 ± 0.010^##^	0.139 ± 0.025	0.117 ± 0.013	0.122 ± 0.004	0.115 ± 0.015	0.116 ± 0.016
	Organ coefficient (%)	0.425 ± 0.033	0.479 ± 0.043	0.462 ± 0.055	0.481 ± 0.076	0.492 ± 0.040	0.487 ± 0.021	0.464 ± 0.061	0.47 ± 0.089
	Organ brain coefficient	27.559 ± 2.222	30.433 ± 2.227	28.091 ± 1.858	30.671 ± 5.540	25.616 ± 2.681	26.653 ± 1.356	25.592 ± 3.165	25.265 ± 3.828
Liver	Weight (g)	1.516 ± 0.126	1.554 ± 0.175	1.395 ± 0.179	1.459 ± 0.166	1.308 ± 0.365	1.309 ± 0.125	1.264 ± 0.056	1.200 ± 0.136
	Organ coefficient (%)	5.163 ± 0.2229	5.367 ± 0.392	5.095 ± 0.908	5.044 ± 0.285	5.521 ± 1.564	5.226 ± 0.366	5.105 ± 0.199	4.890 ± 0.325
	Organ brain coefficient	334.891 ± 24.133	341.672 ± 31.887	309.586 ± 37.999	321.205 ± 33.841	286.589 ± 76.929	285.763 ± 15.815	281.927 ± 17.742	261.124 ± 30.327
Spleen	Weight (g)	0.051 ± 0.011	0.068 ± 0.013**	0.058 ± 0.012	0.070 ± 0.012**	0.071 ± 0.058	0.109 ± 0.026	0.072 ± 0.015^##^	0.0821 ± 0.013^#^
	Organ coefficient (%)	0.174 ± 0.035	0.235 ± 0.046**	0.212 ± 0.048	0.244 ± 0.041**	0.302 ± 0.250	0.436 ± 0.098	0.290 ± 0.057^#^	0.334 ± 0.037^#^
	Organ brain coefficient	11.340 ± 2.571	14.944 ± 2.889*	12.888 ± 2.589	15.512 ± 2.764	15.609 ± 12.469	23.815 ± 5.153	16.051 ± 3.501^##^	17.863 ± 2.812^#^
Kidney	Weight (g)	0.393 ± 0.051	0.394 ± 0.038	0.372 ± 0.044	0.380 ± 0.032	0.279 ± 0.022	0.275 ± 0.007	0.258 ± 0.023	0.273 ± 0.033
	Organ coefficient (%)	1.334 ± 0.103	1.361 ± 0.066	1.348 ± 0.074	1.316 ± 0.072	1.175 ± 0.063	1.100 ± 0.040	1.041 ± 0.057***	1.111 ± 0.083
	Organ brain coefficient	86.663 ± 9.620	86.663 ± 7,032	82.619 ± 8.732	83.641 ± 6.449	61.118 ± 4.329	60.164 ± 1.821	57.592 ± 5.603	59.340 ± 7.176
Testis/Ovary	Weight (g)	0.149 ± 0.040	0.171 ± 0.015	0.164 ± 0.022	0.172 ± 0.010	0.015 ± 0.004	0.018 ± 0.003	0.017 ± 0.003	0.016 ± 0.004
	Organ coefficient (%)	0.504 ± 0.130	0.592 ± 0.056	0.595 ± 0.095	0.600 ± 0.065	0.064 ± 0.015	0.071 ± 0.012	0.070 ± 0.011	0.065 ± 0.016
	Organ brain coefficient	32.811 ± 8.677	37.619 ± 3.236	36.254 ± 4.584	37.921 ± 2.342	3.354 ± 0.854	3.872 ± 0.703	3.884 ± 0.791	3.468 ± 1.016
Epididymis/Uterus	Weight (g)	0.074 ± 0.012	0.079 ± 0.015	0.079 ± 0.012	0.081 ± 0.010	0.105 ± 0.035	0.119 ± 0.049	0.108 ± 0.056	0.146 ± 0.060
	Organ coefficient (%)	0.251 ± 0.031	0.271 ± 0.027	0.287 ± 0.036	0.281 ± 0.044	0.445 ± 0.145	0.471 ± 0.181	0.439 ± 0.243	0.589 ± 0.219
	Organ brain coefficient	16.293 ± 2.333	17.288 ± 2.069	17.558 ± 2.538	17.806 ± 2.366	23.113 ± 7.676	25.863 ± 10.226	24.104 ± 12.374	31.826 ± 12.936
Adrenal gland	Weight (g)	0.007 ± 0.001	0.007 ± 0.010	0.007 ± 0.002	0.006 ± 0.001	0.009 ± 0.001	0.009 ± 0.001	0.008 ± 0.001	0.008 ± 0.001
	Organ coefficient (%)	0.020 ± 0.004	0.023 ± 0.005	0.025 ± 0.006	0.022 ± 0.005	0.037 ± 0.003	0.035 ± 0.004	0.033 ± 0.003	0.032 ± 0.003
	Organ brain coefficient	1.329 ± 0.312	1.435 ± 0.314	1.490 ± 0.358	1.359 ± 0.292	1.903 ± 0.222	1.900 ± 0.237	1.795 ± 0.179	1.726 ± 0.205
Brain	Weight (g)	0.453 ± 0.021	0.454 ± 0.013	0.450 ± 0.014	0.454 ± 0.007	0.456 ± 0.008	0.457 ± 0.020	0.449 ± 0.020	0.460 ± 0.012
	Organ coefficient (%)	1.546 ± 0.083	1.574 ± 0.069	1.642 ± 0.133	1.578 ± 0.092	1.926 ± 0.091	1.828 ± 0.052	1.816 ± 0.124	1.884 ± 0.134

Note: The data were expressed as mean ± SD. “*“or“**” indicates a statistically significant difference at *p* < 0.05 and *p* < 0.01 when compared to the control group; “#“,“##” or,“###” indicates a statistically significant difference at *p* < 0.05, *p* < 0.01 or *p* < 0.001 when compared to the mock group;

Organ coefficient (%) = g organ weight/g body weight×100%; Organ brain coefficient = g organ weight/g brain weight.

Bone marrow smears at D92 showed active bone marrow proliferation in both the mock and BD211 groups, with a significant increase in the proportion of lymphocytes and a relative decrease in the number of granulocytes and erythrocytes, consistent with the trends observed in hematological changes (Supplementary Table S3).

## 4 Discussion

Zynteglo, the first FDA-approved gene therapy for β-thalassemia (August 2022), employs lentiviral-mediated βA-T87Q-globin gene addition to restore functional β-globin production in hematopoietic stem cells (HSCs) – a mechanism shared by BD211. While clinical trials of Zynteglo demonstrate promising transfusion independence rates, its preclinical characterization in animal models remains unpublished, limiting mechanistic insights into biodistribution and long-term safety. This data gap extends to most HSC-based gene therapies currently in development, which predominantly report clinical rather than preclinical findings. The systematic evaluation of BD211 in NCG-X murine models addresses this critical need, providing empirical evidence of stable transgene integration, erythroid-specific expression, and absence of genotoxicity signals. This preclinical evidence not only supports the IND application of BD211 but also establishes a benchmark for comparative safety assessments of lentiviral therapies, particularly regarding insertional mutagenesis risks in immunodeficient models.

NCG-X mice, derived from NCG severely immunodeficient mice, have been genetically edited to introduce a W41 point mutation in the c-Kit gene (Mcintosh et al., 2015). They exhibit deficiencies in T/B/NK cell immunity and suppressed hematopoietic stem cell function, enabling the reconstitution of higher levels of erythroid cells. This makes them an excellent model for human HSCT and the study of thalassemia (Bao et al., 2021). Under the experimental conditions used in this study, after 8 and 13 weeks of administration, the bone marrow of mice in the mock and BD211 groups showed a significant increase in erythroid cells, primarily characterized by a high proportion of immature erythrocytes (hCD235a^+^hCD71^+^). This indicates a dynamic process of erythroid maturation and differentiation. Additionally, widespread expression of β-globin mRNA and β^A−T87Q^-globin mRNA was observed in the bone marrow. The cell classification results from the bone marrow and spleen also demonstrated a significant increase in various subtypes of cells derived from human hematopoietic stem cells, including hCD3^+^, hCD13^+^, hCD19^+^, hCD34^+^, and hCD56^+^. These findings suggest that BD211 has successfully engrafted in the mouse bone marrow, reconstituting the human erythroid system within the mice and differentiating stably into various stages of human erythroid cells. Furthermore, during the directed differentiation process, it has expressed human erythroid-specific genes and the target gene of interest.

During the trial period, mortality or near-death events occurred in all groups, with proportions of 17.50%, 19.23%, 11.54%, and 14.10%, respectively, showing no dose-dependent relationship. Most animals with unclear causes of near-death or death exhibited a mild to moderate increase in bone marrow (sternum) cell numbers, accompanied by neutrophilia. Combining the results of bone marrow smears and hematological examinations at D92, we analyzed that the cause of animal deaths may be related to the implantation of human hematopoietic stem cells. While reconstituting the human erythroid system, this implantation could potentially affect the hematopoietic function of the NCG-X mouse’s bone marrow stem cells, leading to exacerbated anemia. The animal supplier provided data also indicated that the natural mortality rate of these mice begins to increase at 5 weeks of age, reaching up to 30% by 25 weeks, which is significantly higher than that of ordinary NCG mice (Data not published). Moreover, no reports of treatment-related deaths have been found in the safety studies of BD211 or other gene therapies for TDT (Mirza et al., 2025; Kwiatkowski et al., 2024). Therefore, the cause of death in these near-death/dead animals is mainly associated with the spontaneous anemia or immunodeficiency of the model mice rather than BD211.

After 13 weeks of administration (D92), hematological indicators in the mock and BD211 groups showed a decrease in absolute values compared to the solvent control group. This included reductions in red blood cell indices (RBC, HGB, HCT, MCHC, and RET), white blood cell indices (WBC, NEUT, LYMPH, MONO), and platelets (PLT). The decrease was dose-dependent with respect to the human CD34^+^ hematopoietic stem cell dose. Bone marrow smears at D92 also indicated active bone marrow proliferation in the mock control and BD211 groups, with a significant increase in the proportion of lymphocytes and a relative decrease in the number of granulocytes and erythrocytes, consistent with hematological changes. However, in our previous assessment of adverse events following BD211 transplantation, an increase in WBC and lymphocyte counts was observed in patients after intravenous infusion, accompanied by the recovery of other subtypes of cells and stabilization within the normal range (Li et al., 2024). The opposite results observed in animals may be due to the engraftment of human CD34^+^ hematopoietic stem cells in NCG-X mice, which can differentiate into various human cell types, leading to a relatively lower proportion of mouse-derived cells. Additionally, NCG-X mice retain some immune function, and their monocytes (macrophages) in the peripheral blood can continuously phagocytose xenogeneic human terminal blood cells that are released into the mouse peripheral blood after the reconstruction and differentiation of human CD34^+^ hematopoietic stem cells. These changes are attributed to the engraftment, reconstitution, and differentiation of human CD34^+^ hematopoietic stem cells in NCG-X mice rather than specific changes induced by the BD211.

The no observed adverse effect level of intravenously administered BD211 in NCG-X mice was 1.2 × 10^6^ cells per mouse. Subsequently, comprehensive safety studies were conducted on BD211, which supported the application for an Investigational New Drug (IND) clinical trial. The application for “BD211 autologous CD34^+^ hematopoietic stem cell injection” was granted implied permission by the China National Drug Administration (acceptance number: CXSL2300710). The indication for this treatment is TDT.

## Data Availability

The original contributions presented in the study are included in the article/Supplementary Material, further inquiries can be directed to the corresponding authors.
