# Supplementary material for: Preclinical efficacy and safety evaluation of BD211 autologous CD34+ hematopoietic stem cell injection for transfusion-dependent β-thalassemia in NCG-X mice
Source: Front Cell Dev Biol. 2025 Jun 16;13:1607707. doi: 10.3389/fcell.2025.1607707 (PMC12206737; doi:10.3389/fcell.2025.1607707)

Supplementary Table 1. Blood biochemical analysis of NCG-X mice treated with BD211 on D92. Blood samples were analyzed within 8 hours at room temperature using the Model 7180 automated biochemical analyzer.

Parameter	Male mice				Female mice			
	Control	Mock	Low dose	High dose	Control	Mock	Low dose	High dose
Number of animals	9	11	12	11	8	4	9	7
AST (U/L)	61.52 ± 9.11	56.35 ± 7.15	72.18 ± 25.54	61.88 ± 7.32	75.90 ± 22.38	64.88 ± 4.82	64.80 ± 6.62	73.89 ± 5.95
ALT (U/L)	38.34 ± 7.37	38.19 ± 8.81	39.21 ± 19.71	37.84 ± 4.09	35.34 ± 8.65	40.48 ± 3.69	34.11 ± 3.79	38.91 ± 6.71
GGT (U/L)	0.20 ± 0.14	0.08 ± 0.10	0 ± 0	0.07 ± 0.06	0.20 ± 0.20	0.30 ± 0.10	0.06 ± 0.05$^{\#\#}$	0.10 ± 0
CK (U/L)	66.7 ± 19.5	146.9 ± 225.6	97.1 ± 49.6	93.8 ± 47.9	77.5 ± 43.8	48.8 ± 5.4	73.3 ± 26.5	73.7 ± 10.4
ALP (U/L)	56.69 ± 8.19	53.59 ± 5.52	61.58 ± 11.55	60.89 ± 8.11	76.53 ± 21.42	65.05 ± 7.57	91.16 ± 11.56$^{\#\#}$	81.33 ± 17.41
UREA (mmol/L)	9.813 ± 1.636	10.305 ± 1.241	10.435 ± 2.464	10.994 ± 1.662	10.641 ± 2.063	11.088 ± 1.964	11.571 ± 1.588	11.674 ± 1.246
CREA (μmol/L)	2.57 ± 2.73	1.78 ± 1.23	6.37 ± 7.44	1.60 ± 0.46	1.84 ± 1.61	0.30 ± 0	2.47 ± 2.17	2.10 ± 1.13
T.P (g/L)	44.27 ± 2.32	45.51 ± 1.93	46.61 ± 2.81	44.77 ± 1.76	45.60 ± 2.54	42.03 ± 3.52	43.88 ± 2.16	45.07 ± 0.88
ALB (g/L)	28.34 ± 1.95	29.78 ± 0.84	29.53 ± 1.00	29.24 ± 1.31	30.14 ± 2.92	28.50 ± 2.01	29.78 ± 1.24	30.41 ± 0.75
GLB (g/L)	15.92 ± 1.21	15.73 ± 1.35	17.08 ± 3.35	15.54 ± 0.71	15.46 ± 1.70	13.53 ± 1.53	14.10 ± 1.05	14.66 ± 0.25
A/G	1.789 ± 0.168	1.904 ± 0.141	1.782 ± 0.296	1.884 ± 0.087	1.975 ± 0.308	2.115 ± 0.100	2.118 ± 0.111	2.075 ± 0.050
GLU (mmol/L)	6.83 ± 1.61	8.46 ± 1.42*	6.94 ± 1.17$^{\#}$	7.65 ± 1.20	6.48 ± 1.42	7.50 ± 1.34	7.52 ± 1.20	5.66 ± 0.82$^{\#}$
T.BIL (umol/L)	9.74 ± 2.52	13.89 ± 2.48*	11.13 ± 3.57	11.04 ± 3.42	10.55 ± 5.22	12.88 ± 3.41	10.26 ± 4.31	11.17 ± 2.14
TC (mmol/L)	1.487 ± 0.333	1.475 ± 0.203	1.448 ± 0.264	1.431 ± 0.209	1.204 ± 0.138	1.280 ± 0.146	1.188 ± 0.200	1.211 ± 0.183
TG (mmol/L)	0.538 ± 0.160	0.666 ± 0.160	0.475 ± 0.097$^{\#\#}$	0.600 ± 0.117	0.510 ± 0.139	0.585 ± 0.213	0.563 ± 0.197	0.539 ± 0.115
Na^+ (mmol/L)	156.6 ± 1.2	155.0 ± 1.2*	155.5 ± 1.9	156.8 ± 0.9$^{\#\#}$	155.3 ± 1.2	154.3 ± 1.0	154.2 ± 1.4	154.9 ± 1.1
K^+ (mmol/L)	5.508 ± 0.585	5.416 ± 0.238	5.637 ± 0.736	5.404 ± 0.283	5.410 ± 0.351	5.320 ± 0.185	5.303 ± 0.376	5.581 ± 0.423
Cl^- (mmol/L)	116.8 ± 3.0	115.3 ± 1.4	116.6 ± 2.4	117.3 ± 1.4$^{\#}$	116.0 ± 1.6	116.3 ± 2.9	115.7 ± 1.5	117.1 ± 1.3
LDH(U/L)	886.3 ± 351.7	669.5 ± 102.6	883.8 ± 361.1	844.5 ± 290.0	767.8 ± 385.4	544.3 ± 124.1	767.7 ± 248.7	927.9 ± 231.7$^{\#}$

Note: The data were expressed as mean ± SD. "*"or"**" indicates a statistically significant difference at $p < 0.05$ and $p < 0.01$ when compared to the control group; "#","##" or,"###" indicates a statistically significant difference at $p < 0.05$, $p < 0.01$ or $p < 0.001$ when compared to the mock group; Abbreviation: AST, aspartate aminotransferase; ALT, alanine aminotransferase; GGT, γ-glutamyl transpeptidase; CK, creatine phosphokinase; ALP, alkaline phosphatase; CREA, Creatinine; TP, total protein; ALB, albumin; GLB, Globulin; A/G, albumin/globulin ratio; GLU, glucose; BUN, Blood urea nitrogen; T.BIL, total bilirubin; TC, total cholesterol; TG, triglyceride; LDH, lactate dehydrogenase.

Supplementary Table 2. Lymphoid cell subtypes in the peripheral blood of NCG-X mice treated with BD211 on D2, D29, D57, and D92, as analyzed by flow cytometry.

Parameter		Female mice				Male mice			
		Control	Mock	Low dose	High dose	Control	Mock	Low dose	High dose
hCD45$^+$hCD3$^+$in hCD45$^+$/mCD45$^+$ (%)	D2	1.103 ± 0.183	1.120 ± 0.355	0.937 ± 0.270	0.990 ± 0.281	1.133 ± 0.114	1.397 ± 0.416	0.960 ± 0.339	0.707 ± 0.142*#
	D29	0.680 ± 0.059	1.007 ± 0.384	1.807 ± 1.046	0.568 ± 0.263	1.018 ± 0.394	1.077 ± 0.521	1.020	1.033 ± 0.434
	D57	/	0.708 ± 0.167	0.788 ± 0.205	1.123 ± 0.800	/	0.848 ± 0.182	0.905 ± 0.245	1.129 ± 0.205
	D92	/	0.983 ± 0.432	1.405 ± 0.328	1.254 ± 0.448	/	0.957 ± 0.162	1.237 ± 0.084	1.126 ± 0.209
hCD45$^+$hCD3$^+$hCD8$^+$ in hCD45$^+$/mCD45$^+$ (%)	D2	2.053 ± 1.141	1.743 ± 0.588	0.613 ± 0.391	1.165 ± 0.311	1.583 ± 0.915	2.443 ± 0.857	1.547 ± 1.275	2.885 ± 1.701
	D29	0.108 ± 0.066	0.153 ± 0.029	0.370 ± 0.137	0.335 ± 0.251	0.058 ± 0.054	0.200 ± 0.101	0.550	0.482 ± 0.256*
	D57	/	0.098 ± 0.022	0.685 ± 0.221#	0.431 ± 0.170#	/	0.230 ± 0.069	2.115 ± 1.130#	0.911 ± 1.119
	D92	/	0.025 ± 0.010	0.073 ± 0.054	0.108 ± 0.073	/	0.040 ± 0.010	0.130 ± 0.085	0.086 ± 0.050
hCD45$^+$hCD3$^+$hCD4$^+$ in hCD45$^+$/mCD45$^+$ (%)	D2	0.650 ± 0.165	0.883 ± 0.065	0.787 ± 0.515	0.742 ± 0.230	0.435 ± 0.154	1.170 ± 0.165*	0.490 ± 0.151#	0.520 ± 0.220#
	D29	0.658 ± 0.244	1.773 ± 0.564*	1.843 ± 1.160	0.748 ± 0.447	0.445 ± 0.420	1.727 ± 1.080	3.850	1.508 ± 0.979
	D57	/	0.255 ± 0.117	0.820 ± 0.305	0.641 ± 0.392	/	0.605 ± 0.134	1.113 ± 0.191#	0.689 ± 0.164
	D92	/	0.670 ± 0.219	3.350 ± 1.244#	4.298 ± 0.789#	/	1.463 ± 0.383	6.353 ± 1.353#	3.776 ± 1.036#
hCD45$^+$hCD13$^+$ in hCD45$^+$/mCD45$^+$ (%)	D2	0.805 ± 0.148	0.720 ± 0.250	0.627 ± 0.384	0.572 ± 0.170	0.693 ± 0.083	1.440 ± 0.367*	0.647 ± 0.293#	0.498 ± 0.223#
	D29	0.130 ± 0.024	0.840 ± 0.713*	0.860 ± 0.584*	0.462 ± 0.220*	0.278 ± 0.265	0.753 ± 0.506	0.310	0.768 ± 0.446
	D57	/	0.495 ± 0.158	0.568 ± 0.215	0.879 ± 0.545	/	0.883 ± 0.244	0.635 ± 0.189	0.657 ± 0.238
	D92	/	0.540 ± 0.107	0.965 ± 0.362	0.920 ± 0.168#	/	0.687 ± 0.070	0.820 ± 0.106	0.846 ± 0.122
hCD45$^+$hCD19$^+$ in hCD45$^+$/mCD45$^+$ (%)	D2	0.630 ± 0.149	0.627 ± 0.307	0.313 ± 0.168	0.305 ± 0.224	0.445 ± 0.078	1.273 ± 0.388*	0.513 ± 0.410#	0.298 ± 0.063*#
	D29	0.113 ± 0.028	0.557 ± 0.367*	0.893 ± 0.587*	0.568 ± 0.170*	0.145 ± 0.148	0.640 ± 0.470	0.390	1.693 ± 0.804*
	D57	/	1.208 ± 0.269	0.533 ± 0.101	1.064 ± 0.540	/	0.848 ± 0.083	0.458 ± 0.230	0.824 ± 0.432
	D92	/	1.295 ± 0.412	1.413 ± 0.426	1.856 ± 0.517	/	1.317 ± 0.146	0.807 ± 0.304	1.422 ± 0.236
hCD45$^+$hCD56$^+$ in hCD45$^+$/mCD45$^+$ (%)	D2	1.338 ± 0.282	1.423 ± 0.476	0.993 ± 0.273	1.007 ± 0.285	1.210 ± 0.039	2.173 ± 0.376*	1.283 ± 0.342#	0.887 ± 0.248*#
	D29	0.405 ± 0.193	1.023 ± 0.420	1.850 ± 1.402	0.515 ± 0.190#	0.728 ± 0.401	1.010 ± 0.604	1.970	1.088 ± 0.693
	D57	/	0.365 ± 0.070	0.810 ± 0.431	1.001 ± 0.958	/	0.605 ± 0.099	1.045 ± 0.198	0.890 ± 0.328
	D92	/	0.675 ± 0.360	1.518 ± 0.374#	1.448 ± 0.361#	/	0.733 ± 0.125	1.657 ± 0.179#	1.180 ± 0.226#
hCD45$^+$hCD3$^+$hCD4$^+$ /hCD45$^+$hCD3$^+$hCD8$^+$(%)	D2	0.385 ± 0.216	0.533 ± 0.132	1.587 ± 0.954	0.660 ± 0.212	0.350 ± 0.258	0.500 ± 0.095	0.427 ± 0.196	0.242 ± 0.164
	D29	7.318 ± 2.824	11.310 ± 1.852	4.850 ± 2.443#	3.898 ± 3.532#	3.500 ± 3.279	12.100 ± 10.025	7.000	3.360 ± 1.444
	D57	/	2.678 ± 0.999	1.268 ± 0.447	1.599 ± 0.894	/	3.068 ± 1.914	0.625 ± 0.318#	1.289 ± 0.607#
	D92	/	29.675 ± 15.809	106.328 ± 145.820	52.214 ± 27.040	/	37.670 ± 11.929	64.943 ± 36.077	68.820 ± 63.275

Note: The data were expressed as mean ± SD. "*"or"**" indicates a statistically significant difference at $p < 0.05$ and $p < 0.01$ when compared to the control group; "#","##" or,"###" indicates a statistically significant difference at $p < 0.05$, $p < 0.01$ or $p < 0.001$ when compared to the mock group. The percentages indicate the proportion of cells expressing the specified antigens. For gating strategy details, refer to the Methods section. h: human; m: mouse; hCD45/mCD45$^+$ indicates the presence of either human CD45$^+$ cells or mouse CD45$^+$ cells.

Supplementary Table 3. Bone marrow smear of NCG-X mice treated with BD211

Parameter	Male mice				Female mice			
	**Control**	**Mock**	**Low dose**	**High dose**	**Control**	**Mock**	**Low dose**	**High dose**
Number of animals	9	11	12	11	8	4	9	8
NRBC	1.847 ± 0.373	1.806 ± 0.625	2.024 ± 1.860	1.551 ± 0.655	1.932 ± 0.731	2.004 ± 0.540	2.305 ± 0.836	2.206 ± 0.267
Granulocyte Lineage Ratio	58.389 ± 4.595	36.000 ± 10.668***	49.333 ± 9.882*$^{\#\#}$	34.500 ± 5.491***	56.313 ± 8.660	37.000 ± 3.629***	38.111 ± 6.646***	42.188 ± 8.124**
Myeloblast	1.056 ± 0.391	0.682 ± 0.462	1.167 ± 0.537	1.045 ± 0.568	0.875 ± 0.354	0.625 ± 0.250	0.778 ± 0.363	0.750 ± 0.267
Promyelocyte	1.056 ± 0.464	1.909 ± 1.921	1.083 ± 0.597	1.545 ± 0.650	1.125 ± 0.582	1.375 ± 0.629	1.500 ± 0.500	1.188 ± 0.753
Neutrophilic myelo-cyte	5.389 ± 2.133	9.864 ± 2.420***	5.417 ± 1.820$^{\#\#\#}$	7.273 ± 2.875$^{\#}$	5.500 ± 1.793	10.875 ± 4.871	7.333 ± 1.000*	8.688 ± 2.963*
Neutrophilic metamyelocyte	16.667 ± 3.354	9.545 ± 2.307***	14.792 ± 4.137$^{\#\#\#}$	8.727 ± 1.979***	15.688 ± 3.826	10.500 ± 0.707*	9.556 ± 1.333***	10.938 ± 2.884**
Neutrophilic granulocyte band form	30.389 ± 2.219	11.591 ± 9.648***	24.500 ± 4.995$^{\#\#\#}$	13.091 ± 4.300***	30.063 ± 5.361	11.125 ± 2.689	15.611 ± 5.957	17.438 ± 6.806
Neutrophilic segmented granulocyte	2.944 ± 1.184	0.955 ± 1.405**	2.083 ± 1.346$^{\#}$	1.000 ± 0.447**	2.625 ± 2.013	0.750 ± 0.645	2.056 ± 1.130	1.688 ± 0.843
Acidic granulocytes	0.111 ± 0.220	0.545 ± 0.568	0.167 ± 0.326	0.455 ± 0.472	0.125 ± 0.231	0.375 ± 0.750	0.333 ± 0.354	0.313 ± 0.372
Eosinophilic metamyelocyte	0.389 ± 0.417	0.636 ± 0.552	0.083 ± 0.195*$^{\#\#}$	0.955 ± 0.522*	0.188 ± 0.259	1.000 ± 0.000**	0.833 ± 0.500**	0.563 ± 0.563
Eosinophilic granulocyte band form	0.389 ± 0.333	0.273 ± 0.344	0.042 ± 0.144**	0.273 ± 0.261	0.125 ± 0.231	0.375 ± 0.25	0.111 ± 0.220	0.625 ± 0.354**
Eosinophilic granulocyte segmented form	0 ± 0	0 ± 0	0 ± 0	0.136 ± 0.234	0 ± 0	0 ± 0	0 ± 0	0 ± 0
Basophilic myelocyte	0 ± 0	0 ± 0	0 ± 0	0 ± 0	0 ± 0	0 ± 0	0 ± 0	0 ± 0
Basophilic meta-myelocyte	0 ± 0	0 ± 0	0 ± 0	0 ± 0	0 ± 0	0 ± 0	0 ± 0	0 ± 0
Basophilic granulocyte band form	0 ± 0	0 ± 0	0 ± 0	0 ± 0	0 ± 0	0 ± 0	0 ± 0	0 ± 0

Basophilic segmented granulocyte	0 ± 0	0 ± 0	0 ± 0	0 ± 0	0 ± 0	0 ± 0	0 ± 0	0 ± 0
Red Blood Cell Lineage Ratio	32.389 ± 4.379	21.227 ± 5.729***	25.958 ± 5.675*	24.636 ± 7.403*	31.063 ± 6.299	19.250 ± 6.299**	17.667 ± 4.031***	19.250 ± 3.864***
Pronormoblast	1.056 ± 0.464	1.136 ± 0.674	1.417 ± 0.597	1.409 ± 0.995	0.875 ± 0.443	1.500 ± 0.443	0.833 ± 0.354	0.813 ± 0.458
Basophilic erythroblast	3.222 ± 0.667	1.727 ± 0.876**	3.667 ± 1.467###	2.045 ± 0.757*	3.625 ± 1.026	2.250 ± 1.026	1.611 ± 0.894*	2.323 ± 1.889
Polychromatophilic erythroblast	11.111 ± 2.421	6.500 ± 2.480***	9.625 ± 2.586#	7.636 ± 2.721*	12.000 ± 2.053	6.125 ± 2.053**	5.500 ± 1.250***	6.750 ± 2.891***
Normoblast	17.000 ± 3.202	11.864 ± 3.355**	11.250 ± 2.641**	13.545 ± 5.012	14.563 ± 4.484	9.375 ± 4.484*	9.722 ± 2.476*	9.375 ± 1.996**
Lymphocyte Lineage Ratio	4.889 ± 2.028	39.955 ± 11.746***	20.875 ± 9.902***###	37.455 ± 7.738***	8.813 ± 6.458	40.500 ± 4.163***	40.389 ± 7.553***	35.438 ± 10.831***
Prolymphocyte	0 ± 0	0.545 ± 0.688	0.208 ± 0.450	0.727 ± 1.232	0 ± 0	0.125 ± 0.250	0.444 ± 0.464**	0.250 ± 0.267
Lymphocyte	4.889 ± 2.028	39.409 ± 11.655***	20.667 ± 9.701***###	36.727 ± 7.708***	8.813 ± 6.458	40.375 ± 3.966***	39.944 ± 7.654***	35.188 ± 10.872***
Mononuclear Cell Lineage Ratio	4.111 ± 0.993	2.636 ± 0.778**	3.333 ± 1.052	2.955 ± 0.688*	3.688 ± 1.163	3.250 ± 1.190	3.500 ± 0.791	3.000 ± 1.389
Monoblast	0 ± 0	0 ± 0	0 ± 0	0 ± 0	0 ± 0	0 ± 0	0 ± 0	0 ± 0
Monocyte	4.111 ± 0.993	2.636 ± 0.778**	3.333 ± 1.052	2.955 ± 0.688*	3.688 ± 1.163	3.250 ± 1.190	3.500 ± 0.791	3.000 ± 1.389
Other Cell Lineage Ratio	0.222 ± 0.363	0.182 ± 0.337	0.500 ± 0.426	0.455 ± 0.522	0.125 ± 0.231	0 ± 0	0.333 ± 0.354	0.125 ± 0.231
Plasma cell	0 ± 0	0 ± 0	0 ± 0	0.045 ± 0.151	0 ± 0	0 ± 0	0 ± 0	0 ± 0
Phagocyte	0.222 ± 0.363	0.182 ± 0.337	0.500 ± 0.426	0.318 ± 0.405	0.125 ± 0.231	0 ± 0	0.333 ± 0.354	0.125 ± 0.231
Reticular cell	0 ± 0	0 ± 0	0 ± 0	0 ± 0	0 ± 0	0 ± 0	0 ± 0	0 ± 0
Mast Cell	0 ± 0	0 ± 0	0 ± 0	0.091 ± 0.302	0 ± 0	0 ± 0	0 ± 0	0 ± 0
Unclassified cell	0 ± 0	0 ± 0	0 ± 0	0 ± 0	0 ± 0	0 ± 0	0 ± 0	0 ± 0
Osteoblast	0 ± 0	0 ± 0	0 ± 0	0 ± 0	0 ± 0	0 ± 0	0 ± 0	0 ± 0
Osteoclast	0 ± 0	0 ± 0	0 ± 0	0 ± 0	0 ± 0	0 ± 0	0 ± 0	0 ± 0
Other	0 ± 0	0 ± 0	0 ± 0	0 ± 0	±	0 ± 0	0 ± 0	0 ± 0

Note: The data were expressed as mean ± SD. "*"or"**" indicates a statistically significant difference at $p < 0.05$ and $p < 0.01$ when compared to the control group; "#","##" or,"###" indicates a statistically significant difference at $p < 0.05$, $p < 0.01$ or $p < 0.001$ when compared to the mock group; Abbreviation: NRBC, neutrophil to red blood cell ratio.

Supplementary Table 4. List of primer sequences employed in qRT-PCR analysis

Gene	Sequence
wild-type β^{A}-globin-F	TCAAGGGCACCTTTGCCACA
wild-type β^{A}-globin-R	AATTCTTTGCCAAAGTGATGGG
$\beta^{A\text{-}T87Q}$-globin-F	TCAAGGGCACCTTTGCCCAG
$\beta^{A\text{-}T87Q}$-globin-R	AATTCTTTGCCAAAGTGATGGG
GAPDH-F	ACCCACTCCTCCACCTTTGA
GAPDH-R	CTGTTGCTGTAGCCAAATTCGT
Lentiviral WPRE-F	GGCACTGACAATTCCGTGGT
Lentiviral WPRE-R	AGGGACGTAGCAGAAGGACG
Lentiviral WPRE-Probe	5'FAM- ACGTCCTTTCCATGGCTGCTCGC-3'MGB
RNase P-F	AGATTTGGACCTGCGAGCG
RNase P-R	GAGCGGCTGTCTCCACAAGT
RNase P-Probe	5'VIC-TTCTGACCTGAAGGCTCTGCGCG-3'BHQ1

Note: qRT-PCR, quantitative reverse transcription polymerase chain reaction; GAPDH, glyceraldehyde 3-phosphate dehydrogenase;

Supplementary Table 5. List of reagents.

Reagent Name	Manufacturer	Batch No./Catalog No.
StemSpan SFEM II Serum-Free Medium for Hematopoietic Stem Cells	STEMCELL	9655
X-VIVO 15 Serum-Free Medium With Gentamicin and Phenol Red	Lonza	04-418Q
StemSpan™ Erythroid Expansion Supplement (100X)	STEMCELL	2692
Hemoglobin β Antibody (37-8) FITC	Santa Cruz Biotechnology	sc-21757
MagaBio plus Universal Genomic DNA Purification Kit II	BioFlux	BSC74L1E/ BSC74M1E
2 × AceQ Universal U+ Probe Master Mix V2	Vazyme	Q513-02
MagaBio plus Total RNA Purification Kit II	BioFlux	BSC69L1E/ BSC69M1E
Blood Total RNA Kit	HANGZHOU SIMGEN BIOTECHNOLOGY	5201050
2 × SuperReal PreMix Plus (SYBR Green)	TIANGEN	FP205-02
HiScript III All-in-one RT SuperMix Perfect for qPCR	Vazyme	7E740A3
MethoCult H4435 Enriched Medium	STEMCELL	4435
IMDM with 25 mM HEPES	STEMCELL	36150
V-PLEX® and V-PLEX PLUS Proinflammatory Panel 1 (human) Kit	Meso Scale Discovery	K15049G
PerCP/Cyanine5.5 anti-mouse CD45	Biolegend	103132
PE anti-human CD45	Biolegend	304039
APC anti-human CD3	Biolegend	300312
Zombie Aqua™ Fixable Viability Kit	Biolegend	423111
Brilliant Violet 510™ anti-human CD4	Biolegend	317444
APC/Fire™ 750 anti-human CD8	Biolegend	344746
PE/Cyanine7 anti-human CD19	Biolegend	302216
PE/Dazzle 594 anti-human CD56(NCAM)	Biolegend	362544
Brilliant Violet 605™ anti-human CD13	Biolegend	301728
Lysing Buffer	BD	555899
DPBS basic （1X）	Gibco	C14190500BT
Brilliant Violet 421™ anti-human CD34	Biolegend	343610
PerCP/Cyanine5.5 anti-human CD45	Biolegend	304028
APC anti-human CD71	Biolegend	334108
PE anti-human CD235a	Biolegend	349106
True-Nuclear Transcription Factor Buffer Set	BioLegend	424401
APC anti-human CD71 Antibody	BioLegend	334108
PE anti-human CD235a (Glycophorin A) Antibody	BioLegend	349106

Supplementary Table 6. List of instruments.

Equipment Name	Manufacturer	Model
Fluorescent cell counter	Nexcelom Bioscience	Cellometer K2
Flow Cytometer	Beckman Coulter	DxFlex B5-R3-V3
Fully Automatic Hematology Analyzer	Sysmex	XN-1000V (B1)
Fully Automatic Biochemical Analyzer	Hitachi	BS-820/7180
Real-time Fluorescent Quantitative PCR System	Thermo Fisher Scientific	QuantStudio 5
Fluorescent Quantitative PCR System	Analytik jena	qTOWER3 G
Fully Automatic Nucleic Acid Extraction and Purification Instrument	BioFlux	NPA-96
MESO QuickPlex SQ 120MM	Meso Scale Discovery	SQ 120MM
High-throughput Nanodrop Spectrophotometer	IMPLEN	N120

Supplementary Figure 1. Representative gating strategies for cell differentiation populations in peripheral blood.

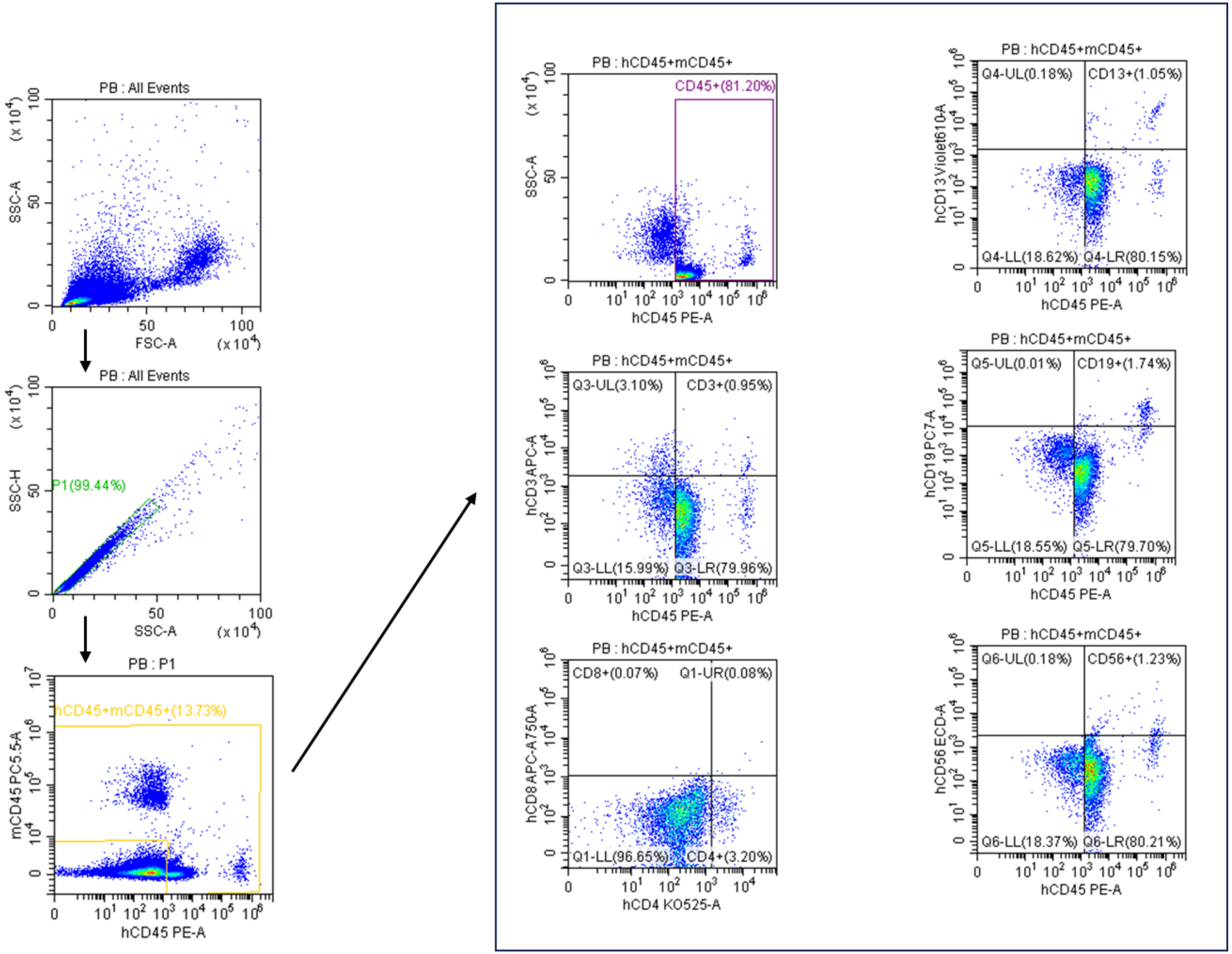

Supplementary Figure 2. Representative gating strategies for cell differentiation populations in spleen.

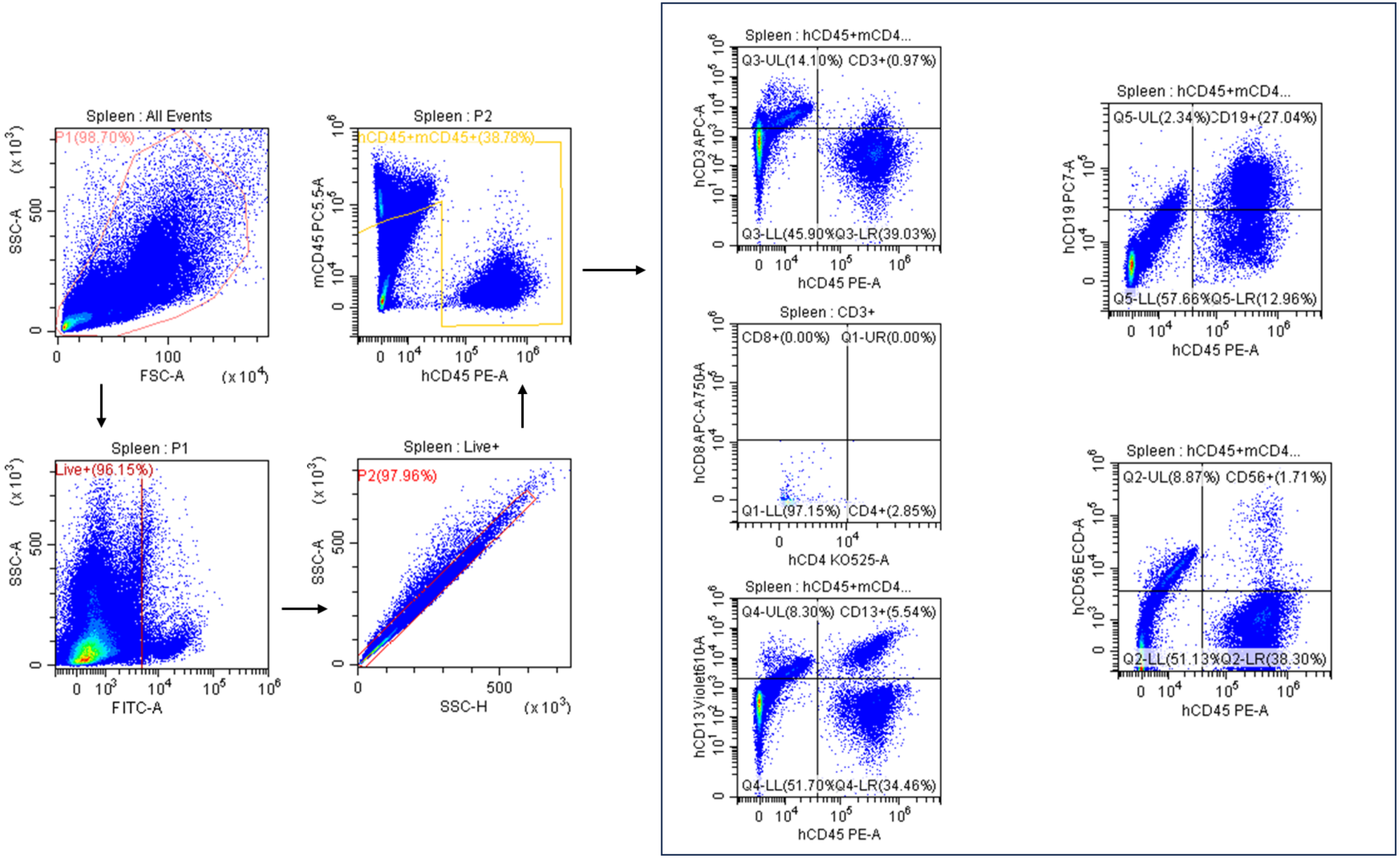

Supplementary Figure 3. Representative gating strategies for cell differentiation populations in bone marrow.

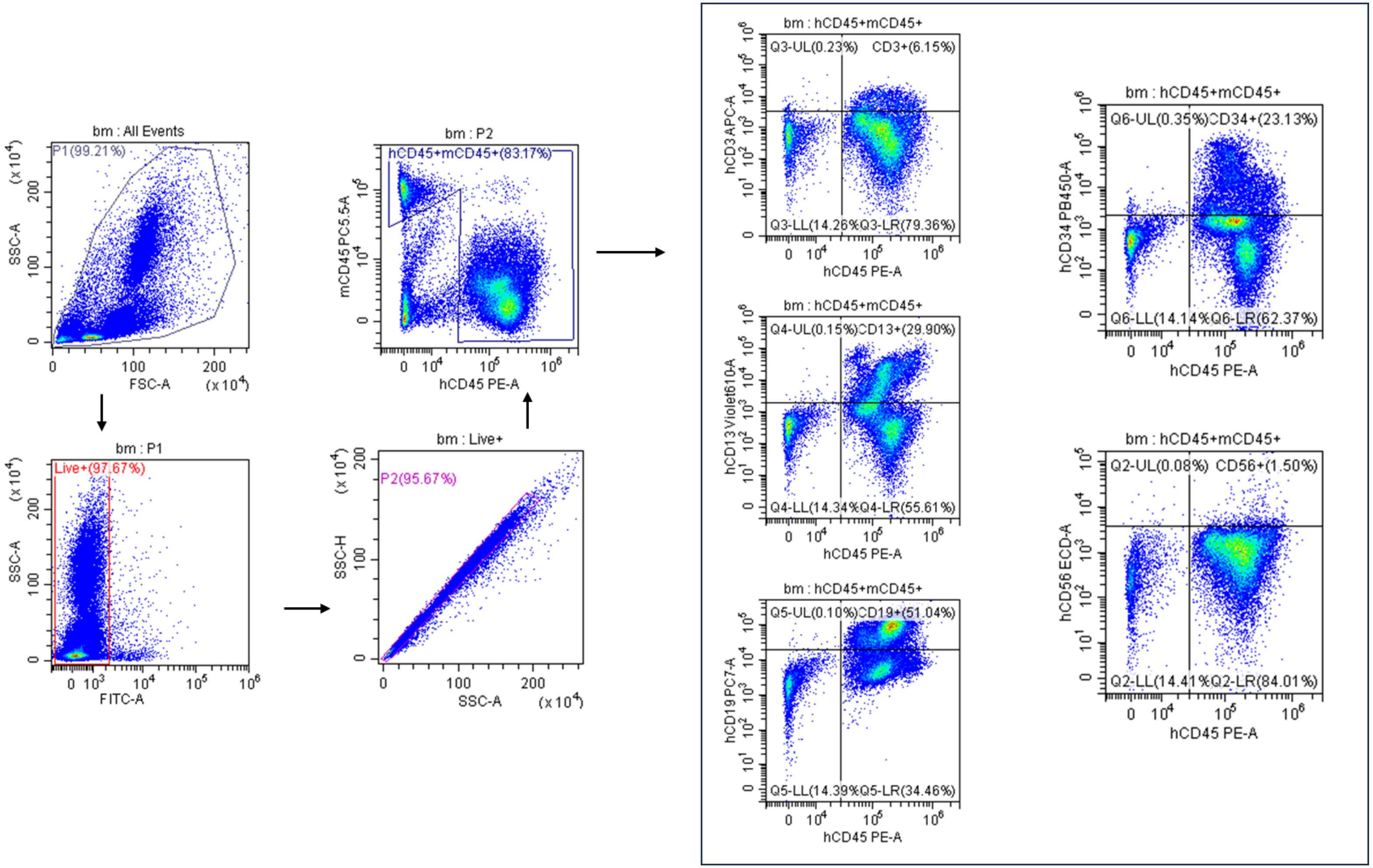

Supplementary Figure 4. Representative gating strategies for erythroid maturation and differentiation in peripheral blood.

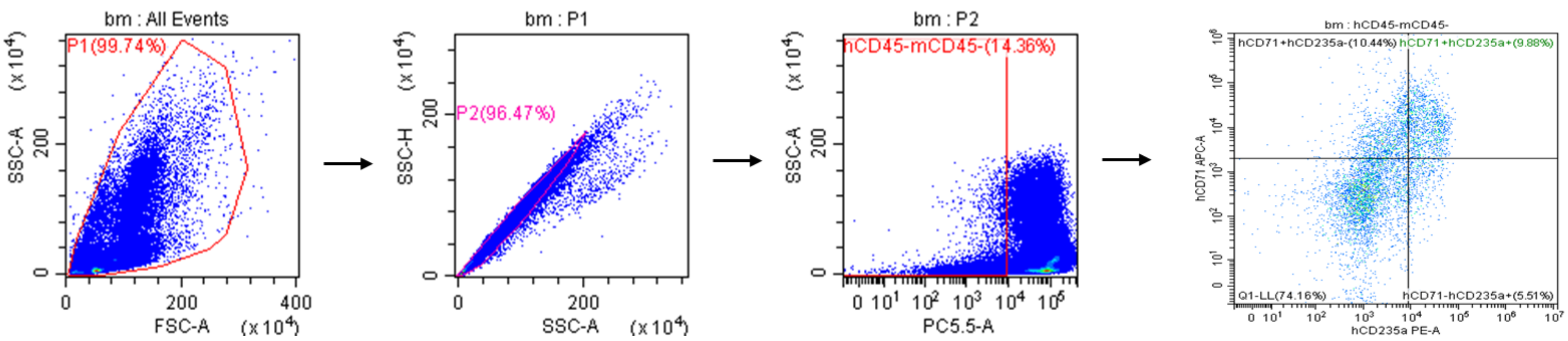

Supplement: Supplementary file 1 [file DataSheet1.pdf]
